# Arts and Cultural Engagement, Reportedly Antisocial or Criminalized Behaviors, and Potential Mediators in Two Longitudinal Cohorts of Adolescents

**DOI:** 10.1007/s10964-022-01591-8

**Published:** 2022-03-23

**Authors:** Jessica K. Bone, Feifei Bu, Meg E. Fluharty, Elise Paul, Jill K. Sonke, Daisy Fancourt

**Affiliations:** 1 Research Department of Behavioural Science and Health, Institute of Epidemiology & Health Care, University College London, London, UK; 2 Center for Arts in Medicine, University of Florida, Gainesville, Florida US

**Keywords:** Arts, Cultural engagement, Delinquency, Delinquent attitudes, Self-control, Longitudinal

## Abstract

Arts and cultural engagement is a potential strategy for reducing or preventing reportedly antisocial or criminalized behaviors (those previously and problematically termed as “delinquent”) in adolescence. However, most research to date has focused on arts-based interventions and has not tested arts and cultural engagement in large population-based longitudinal studies. This study investigated whether arts and cultural engagement reduced reportedly antisocial or criminalized behaviors in two large nationally representative cohorts, the National Longitudinal Study of Adolescent to Adult Health (*n* = 10,610; 50% female, 72% White, age range = 11–21 mean = 15.07) and the National Education Longitudinal Study of 1988 (*n* = 15,214; 50% female, 73% White, age range = 13–16 mean = 14.38). Structural equation modelling also allowed exploration of two potential mechanisms that might link arts and cultural engagement to reportedly antisocial or criminalized behaviors (self-control and attitudes towards these behaviors). More arts and cultural engagement was associated with fewer reportedly antisocial or criminalized behaviors, better self-control scores, and fewer positive perceptions of reportedly antisocial or criminalized behaviors concurrently and one to two years later. Arts and cultural engagement may provide opportunities for adolescents to realize positive developmental outcomes, reducing their risk of reportedly antisocial or criminalized behaviors.

## Introduction

Adolescence has long been characterized as a period of increased risk taking (Steinberg, 2004). In line with this, school misconduct, substance use, unsafe sex, antisocial, and criminalized behaviors become more common during adolescence (van Nieuwenhuijzen et al., 2009). Reportedly antisocial or criminalized behaviors include a wide range of actions that are counter to accepted behavioral norms or the law, and often include minor crime such as stealing, selling drugs, and using weapons, as well as antisocial behavior like fighting and not following rules. These behaviors have previously and problematically been termed as “delinquent”. Substance use is not included in this definition as it often occurs separately to these behaviors in adolescence (van Nieuwenhuijzen et al., 2009). Although such behaviors are to some extent normative and limited to adolescence (Agnew, 2003), they are negatively associated with health and wellbeing (Walsh et al., 2013) and other health behaviors (Bradshaw et al., 2010). Additionally, if these behaviors become established during adolescence, they may be maintained through adulthood (Cook et al., 2015). Preventing or reducing these behaviors in adolescence, alongside preventing their root causes (such as early life adversity and structural determinants of health), is thus not only important for health and wellbeing during adolescence, but also has wide ranging impacts into adulthood. Arts and cultural engagement is a potential strategy for reducing or preventing reportedly antisocial or criminalized behaviors in adolescence. However, most research to date has focused on arts-based interventions and has not used large nationally representative samples to test whether ubiquitous arts and cultural engagement, as part of everyday life, can reduce subsequent reportedly antisocial or criminalized behaviors. This study thus contributes to the prevention and reduction of reportedly antisocial or criminalized behaviors by examining the extent to which arts and cultural engagement may affect adolescent behavior.

### The Role of Arts and Culture

While the arts are conceptually difficult to define, there are several cross-cultural characteristics recognized as fundamental to art. These include the art being valued in its own right (and not just as a utility), providing imaginative experiences for both the producer and audience, and comprising or provoking an emotional response (Adajian, 2018). Arts and culture are commonly split into ﻿activities that are receptive, involving art that has been created and is now experienced by an audience (e.g., going to museums, galleries, performances), and those that are participatory, requiring creation of and involvement in the arts (e.g., dancing, making music, reading; Fancourt & Finn, 2019). Arts engagement can also encompass broader creative activities that, whilst not always labelled as ‘arts’, share similar properties of creative skill and imagination, such as gardening, cooking, and other hobbies (Fancourt et al., 2021).

A range of arts-based intervention programs have been developed to prevent and reduce reportedly antisocial or criminalized behaviors among adolescents with some success (Hughes, 2005). For example, arts education programs consisting of drama (Belliveau, 2005) or visual arts (Bickley-Green, 2007) have been used to reduce bullying in schools. Additionally, a group drumming intervention was found to reduce aggressive and violent behaviors and absences from school (Wood et al., 2013). In another study, an expressive writing intervention reduced aggressive behavior and emotional lability (Kliewer et al., 2011). A national evaluation of arts-based programs in the US, which involved participatory arts such as drama, photography, and dance, provided preliminary evidence that they reduced reportedly antisocial or criminalized behavior (Clawson & Coolbaugh, 2001). In these arts programs, adolescents may gain opportunities to improve their communication skills, self-confidence (Hughes, 2005), problem-solving skills, sense of purpose and autonomy, and social competence (Grigorenko, 2020), as well as finding new ways for positive self-expression (Gussak & Ploumis-Devick, 2004). The arts may thus have an important role in changing the individual, institutional, and social circumstances that can lead to reportedly antisocial or criminalized behaviors. Yet, arts-based interventions often involve the identification of adolescents who could benefit from programs designed for “delinquents”, meaning that they may be stigmatizing, and require extensive resources. As a result, such programs may not reach or adequately serve all adolescents in need. Instead, everyday arts and cultural engagement could be seen as a primary prevention strategy, aiming to prevent reportedly antisocial or criminalized behaviors in young people before they emerge.

In line with this, participation in extracurricular activities, including arts and culture as well as sports, academic, and political activities, was found to be associated with reduced “delinquency” in a systematic review (Feldman Farb & Matjasko, 2012). Observational studies have shown that rates of participation in extracurricular activities (including artistic and non-artistic activities) are cross-sectionally associated with lower levels of stealing, destroying public property, fighting (Mahoney & Stattin, 2000), truancy, vandalism (Harrison & Narayan, 2003), breaking school rules (Schmidt, 2003), and reportedly antisocial and criminalized behaviors overall (Rose-Krasnor et al., 2006). There is also some longitudinal evidence that extracurricular activity participation is associated with reduced misconduct two years later (Schmidt, 2003), and less school misbehavior up to three years later (Fleming et al., 2008). Yet, very little research has focused specifically on arts and cultural engagement (Feldman Farb & Matjasko, 2012). At the school level, the proportion of youth who participate in arts activities is cross-sectionally associated with lower levels of reportedly antisocial and criminalized behavior, although there is significant variability across schools (Guest & McRee, 2009). At the individual level, participation in performing and fine arts has been associated with lower rates of skipping school (Eccles & Barber, 1999), dropping out of school (McNeal, 1995), and being arrested (Zill et al., 1995). In contrast, a later study found no evidence that arts participation was associated with reportedly antisocial and criminalized behaviors concurrently or over the subsequent six years (Fauth et al., 2007). These inconsistent findings on the arts could be due to the small samples of most previous studies (*n* < 1000). It thus remains unclear whether arts and cultural engagement can reduce subsequent reportedly antisocial or criminalized behaviors at a population level.

### Potential Mechanisms

Arts and cultural engagement may reduce reportedly antisocial or criminalized behaviors through mechanisms such as altered attitudes towards these behaviors (Clawson & Coolbaugh, 2001), better self-control (Alemán et al., 2017), increased empathy (Kou et al., 2020), more prosocial behavior (Konrath & Kisida, 2021), reduced boredom (Newberry & Duncan, 2001), enhanced emotion regulation (Fancourt & Ali, 2019), and improved self-esteem (Mak & Fancourt, 2019). This study focusses on two of these potential mechanisms, identified as key factors to reduce offending, which are attitudes and self-control (Social Exclusion Unit, 2002). Arts-based interventions for those in the legal system often directly aim to alter young people’s attitudes towards reportedly antisocial or criminalized behaviors, as attitudes are strongly associated with behavior (Hughes, 2005). Arts and cultural engagement may promote more positive attitudes by providing a sense of purpose and achievement, reducing boredom and anger, giving access to a new peer group, and improving social relationships, understanding of others, and prosocial thinking. Attitudes may be easier to change than other trait-like mechanisms (e.g., self-control, empathy) as they are altered by the amount of time spent in criminogenic environments during adolescence (Janssen et al., 2018). In line with this, changing attitudes is an important intermediate outcome of arts programs in the US (Clawson & Coolbaugh, 2001), and community music sessions have been shown to improve the attitudes of young people towards criminalized behaviors (Clennon, 2013). Nevertheless, it is currently unclear whether ubiquitous arts and cultural engagement, which is not a targeted intervention, can alter attitudes towards reportedly antisocial or criminalized behaviors. Exploring this is important as changes in beliefs are likely to translate to changes in behavior, meaning attitudes could mediate the association between arts and cultural engagement and behavior.

Self-control is the ability to regulate one’s emotions, thoughts, and behaviors (Gottfredson & Hirschi, 1990), including the concepts of impulsivity, sensitivity towards others, diligence, and risk-seeking. An inability to regulate thoughts and behaviors and high impulsivity and risk-taking (i.e., low self-control) has been associated with increases in a range of reportedly antisocial or criminalized behaviors in adolescence (Gottfredson & Hirschi, 1990). This includes minor crime, academic fraud, binge drinking, drunk dialing, and profanity (Reisig & Pratt, 2011) as well as measures of overall “delinquency” (Wolfe & Hoffmann, 2016). Although self-control is a relatively stable trait (Britt & Gottfredson, 2011), it may still be modifiable during adolescence, as a range of interventions have successfully improved self-control in childhood and early adolescence (Piquero et al., 2010). These programs also reduce delinquency. Arts and cultural engagement may enhance self-control by providing opportunities for expressing emotions, safe exploration of boundaries, and learning from risk-taking, as well as improving communication, problem-solving, attitudes towards others, and feelings of calm and acceptance (Parker et al., 2018). Studies of arts programs in the legal system (Bilby et al., 2013) and the community (Center for the Study of Art & Community, 2007) have provided qualitative evidence that they can improve self-control. There is also strong quantitative evidence from a randomized trial, which found that participation in national orchestras improved self-control (Alemán et al., 2017), and a meta-analysis demonstrating that singing activities increase self-control (Moon, 2017). Similarly, dance programs can offer opportunities for practicing self-control (Milliken, 2002) and a theater-based intervention enhanced self-control (Farhadi & Tabatabaei Zavareh, 2020). Furthermore, arts and cultural engagement is also associated with enhanced emotion regulation (Fancourt & Ali, 2019), which is closely positively related to self-control (Paschke et al., 2016). It is therefore possible that arts and cultural engagement may prevent or reduce reportedly antisocial or criminalized behaviors through improvements in self-control.

### Importance of Sociodemographic Factors

There is a social gradient in both arts and cultural engagement (Mak & Fancourt, 2021) and reportedly antisocial or criminalized behaviors in adolescence (Shader, 2000), as a range of factors are likely to influence both types of behavior. These factors include adolescents’ age, gender, race/ethnicity, language spoken at home, parental socioeconomic position (as indicated by factors such as education, marital status, and household income), and neighborhood characteristics (e.g., urbanicity). These factors must be assessed to test whether associations between arts and cultural engagement and reportedly antisocial or criminalized behaviors are due to self-selection, or because arts and cultural engagement can lead to changes in outcomes during adolescence (Feldman & Matjasko, 2005). Research must also account for the fact that adolescents spend much of their time at school, and adolescents within schools are more similar to each other than to adolescents at other schools, due to historic and contemporary neighborhood and school segregation.

The associations between arts and cultural engagement and reportedly antisocial or criminalized behaviors may also differ according to sociodemographic factors such as gender (Feldman Farb & Matjasko, 2012). In general, females are more likely to participate in arts and cultural activities (Mak & Fancourt, 2021) and are less likely to engage in reportedly antisocial or criminalized behaviors (Junger-Tas et al., 2004). Although there is evidence that the associations between extracurricular participation, weapon carrying, and fighting differ according to gender (Linville & Huebner, 2005), as do the associations between sports participation and school misconduct (Miller et al., 2005), it remains unclear whether associations specifically with arts and cultural engagement differ according to gender.

## Current Study

Arts and cultural engagement is a potential strategy for reducing or preventing reportedly antisocial or criminalized behaviors in adolescence. However, most research to date has focused on arts-based interventions and has not tested arts and cultural engagement in large population-based longitudinal studies. This study aimed to investigate whether overall arts and cultural engagement (extracurricular arts and creative activities, hobbies, going to museums and concerts) in mid-adolescence influenced reportedly antisocial or criminalized behaviors in mid- to late adolescence. This study also tested two distinct potential mechanisms that might link arts and cultural engagement to reportedly antisocial or criminalized behaviors: attitudes towards these behaviors and self-control. The current study aimed to test three hypotheses. First, that more arts and cultural engagement would lead to fewer reportedly antisocial or criminalized behaviors after accounting for a range of sociodemographic factors (age, gender, race/ethnicity, first language, parental socioeconomic position, neighborhood urbanicity, and school; Hypothesis 1). Second, that this relationship was partially mediated by self-control, which has been shown to be improved by arts and cultural engagement and to be linked to reportedly antisocial or criminalized behaviors (Hypothesis 2). Third, that this relationship was also partially mediated by attitudes towards reportedly antisocial or criminalized behaviors, which are often the target of arts-based interventions and are also associated with behavior (Hypothesis 3). In exploratory sensitivity analyses, this study also aimed to test first whether these associations differed according to gender, and second whether the effects are maintained when focusing only on violent behavior.

## Methods

### Design

In this study, data from two longitudinal nationally representative studies, the National Longitudinal Study of Adolescent to Adult Health (Add Health; Harris et al., 2009) and the National Education Longitudinal Study of 1988 (NELS:88; Curtin et al., 2002), were used to allow comparison across cohorts. These cohorts varied in participant age, time scale, and measures of arts and cultural engagement and reportedly antisocial or criminalized behaviors (RACBs). These cohorts were chosen because of both their similarities and differences; replicating findings across two cohorts that are not directly comparable would suggest that the results are conceptually robust and relevant from mid-adolescence (NELS:88) to young adulthood (Add Health). The cohorts also differed in their inclusion of measures of self-control (Add Health) or attitudes towards RACBs (NELS:88), allowing for comparisons of these processes as mediators of the associations between arts and cultural engagement and RACBs.

### Add Health Cohort

#### Sample

The first sample was drawn from the National Longitudinal Study of Adolescent to Adult Health (Add Health); a longitudinal study of a nationally representative sample of adolescents who were in grades 7–11 (aged 12–18 years) during the 1994–95 school year and have been followed for five waves (Harris et al., 2009). The Add Health restricted-use data were used for this study. Participants who completed waves one (1994–1995), two (1996), and three (2001-2002) of Add Health were eligible for inclusion. At wave one, 20,745 adolescents and 17,670 of their parents participated. Of these adolescents, 14,738 participated at wave two and 15,197 at wave three. A total of 10,828 adolescents completed interviews at waves one, two, and three and also had parental data at wave one (Chen & Mullan Harris, 2020). Participants were excluded due to missing data on school (*n* = 2) and exogenous covariates (*n* = 216), leaving a final sample of 10,610 adolescents.

#### Measures

Arts and cultural engagement, RACBs, and self-control were modelled as latent constructs using the indicator variables described below. On each latent factor, higher scores indicated more arts and cultural engagement, more RACBs, worse self-control, or lower socioeconomic position. See the Supplementary Materials for full details on questions, scoring, and loadings on the latent variables (Table S1 and Fig. S1).

##### Arts and cultural engagement

Engagement in a range of activities was measured at wave one with ten questions (Table S1). Adolescents were asked whether they were participating, or planned to participate, in any of the following school clubs, organizations, or teams: book club; drama club; band; cheerleading/dance; chorus/choir; orchestra; or newspaper (all answered yes, no). They were also asked how many times they had done hobbies in the past week (responses collapsed to none, one or more). Finally, participants were asked whether they had been to a movie, play, museum, concert, or sports event with a) their biological or resident mother or b) their biological or resident father in the past four weeks (yes, no). All ten questions were included as indicator variables for the latent construct of arts and cultural engagement.

Arts and cultural engagement was measured as an overarching construct as this offers a more parsimonious way of understanding the role of the arts in youth outcomes (Martin et al., 2013). Separating the many different forms of arts would have presented a complex set of factors that would have been difficult to model. Although the items measuring arts and cultural engagement had low internal consistency (Cronbach’s α = 0.41), this was not surprising. The latent construct included a range of different types of activities, and it is not expected that an adolescent who engages in one activity will also engage in the rest. Using a latent variable approach considers the importance of each activity for the overarching construct of engagement, and also incorporates measurement error into the model (Kline, 2015). Although most items were related to participation in school arts clubs, the questions on family events loaded more highly onto this construct (Fig. S1), so it is likely to give a balanced overall indication of arts and cultural engagement.

##### Reportedly antisocial or criminalized behavior (RACB)

RACB was self-reported by adolescents at waves one to three with a “delinquency” scale developed for Add Health. Eleven items that were measured consistently across waves were included (as done previously; Wilkinson et al., 2019). Five questions about non-violent behaviors and six questions about violent behaviors performed in the past 12 months (never, 1-2 times, 3-4 times, ≥5 times) were combined into one scale (Table S1). Non-violent behaviors were: 1) damage property; 2) steal something worth less than $50; 3) steal something worth more than $50; 4) burglarize a building; 5) sell drugs. Violent behaviors were: 1) seriously injure someone; 2) use or threaten someone with a weapon; 3) take part in group fight; 4) use a weapon in a fight; 5) pull a knife or gun on someone; 6) shoot or stab someone. The overall scale had good to acceptable internal consistency across waves (wave 1 α = 0.78, wave 2 α = 0.78, wave 3 α = 0.69).

##### Self-control

Self-control was self-reported by adolescents at waves one and two with a list of questions that have previously been combined to measure self-control (Beaver et al., 2009, Wolfe & Hoffmann, 2016). Thirteen items were consistently measured at waves one and two, nine of which were included in this study (as indicated by an exploratory factor analysis). Questions were about impulsivity, forward-thinking skills, self-centeredness, and trouble with a range of school-based activities, such as getting along with teachers and other students and paying attention (Table S1). On the latent factor, higher scores indicated worse self-control. The self-control scale had good internal consistency across waves (wave 1 α = 0.72, wave 2 α = 0.72).

##### Covariates

A range of sociodemographic factors were measured at wave one, selected based on their availability in both Add Health and NELS:88. *Adolescent-reported* covariates were age (years), gender (male, female), race/ethnicity (White, Black/African American, Asian/Pacific Islander, Other [including Hispanic, American Indian/Native American, and Other]), first language spoken at home (English, other), and resident parents’ highest level of education (less than high school, high school, some college, college graduate). *Parent-reported* covariates were parental marital status (married, unmarried [including divorced, separated, widowed, and never married]) and household income (quartiles: $0–$20,000, $21,000–$38,000, $39,000–$60,000, >$60,000). Finally, *interviewers reported* the urbanicity of adolescents’ home location (urban, suburban, rural).

### NELS:88 Cohort

#### Sample

The second sample was drawn from the National Education Longitudinal Study of 1988 (NELS:88), a longitudinal study of a nationally representative sample of over 24,000 adolescents who were in 8^th^ grade (aged 14-15 years) during the 1987–88 school year and have been followed for five waves (Curtin et al., 2002). Participants who completed waves one (1988), two (1990), and three (1992) were eligible for inclusion in this study, so the publicly available NELS 1988-1992 data were used (Ingels et al., 1994). This included 24,599 adolescents at wave one, 17,424 of whom were followed-up at wave two, and 16,489 at wave three. At wave one, 22,651 adolescents had complete parental questionnaires. A total of 15,552 adolescents completed waves one to three of NELS:88 and had parental data at wave one. Participants missing data on exogenous covariates (*n* = 338) were excluded, leaving a final sample of 15,214 participants.

#### Measures

Arts and cultural engagement, RACBs, and attitudes towards RACBs were modelled as latent constructs using the indicator variables described below. On each latent factor, higher scores indicated more arts and cultural engagement, more RACBs, more positive perceptions of RACBs, or higher socioeconomic position. See the Supplementary Materials for full details on questions, scoring, and loadings on the latent variables (Table S2 and Fig. S2).

##### Arts and cultural engagement

Engagement in a range of activities was measured at wave one with 16 questions (all answered yes, no; Table S2). Adolescents were asked whether they had participated, or will participate, in any of the following school activities during the current school year: band/orchestra, chorus/choir, dance, drama club, and student newspaper. They were also asked whether they had participated in hobby clubs outside of school that year and whether they read each week on their own outside school (not in connection with schoolwork). Additionally, participants’ parents reported whether their child attended classes outside of their regular school to study: art; music; dance; and the history and culture of their ancestors. Finally, parents reported whether their child: borrows books from the public library; attends concerts or other musical events; goes to art museums; goes to science museums; and goes to history museums. All 16 questions were included as indicator variables for the latent construct of arts and cultural engagement, and these items had acceptable internal consistency (α = 0.66). This latent variable is thus a broader indicator of overall arts and cultural engagement than the measure in the Add Health cohort. The highest factor loadings were for parent reports of whether adolescents went to art or music classes, the library, concerts, or museums (Fig. S2), suggesting that this latent factor may have captured a different dimension of arts and cultural engagement to the items measured in Add Health.

##### Reportedly antisocial or criminalized behavior (RACB)

RACB was self-reported by adolescents in a range of questions at waves one to three. Only questions that were consistent across waves and could be interpreted as measuring RACBs were included (Table S2). Three items measured the frequency of behaviors in the first semester of the current school year, including getting into trouble for not following school rules, parents receiving a warning about behavior at school, and getting into a physical fight at school (never, sometimes, often). It is possible that getting into trouble for behavior at school may not be an RACB, as it could be a result of experiencing a teacher’s loss of temper through no fault of one’s own (Hamre & Pianta, 2006). This could have resulted in biased measurement of RACBs in this study, with RACBs appearing higher in adolescents who were not liked by their teacher, particularly if racial biases influenced the teacher’s behavior (Bryan, 2017). Nonetheless, behavior was self-reported by adolescents, meaning that they determined for themselves what getting into trouble meant. In line with this, the items had good to acceptable internal consistency across waves (wave 1 α = 0.75, wave 2 α = 0.60, wave 3 α = 0.47).

##### Attitudes towards reportedly antisocial or criminalized behaviors (attitudes towards RACBs)

Attitudes were self-reported by adolescents at wave two with 12 items (Table S2). Adolescents were asked how often they felt it was ‘OK’ for them to perform a range of behaviors, including getting into physical fights, belonging to gangs, making racist and sexist remarks, and stealing (never, rarely, sometimes, often). The items measuring these attitudes had good internal consistency (α = 0.81). It is important to note that this scale measured *general attitudes* towards RACBs, whereas the above measure of RACBs was narrower, and measured only *school-based behaviors*.

##### Covariates

Sociodemographic factors were measured at wave one. *Adolescent-reported* covariates were age (≤13, 14, 15, ≥16 years), gender (male, female), race/ethnicity (White, Black, Asian/Pacific Islander, Other [including Hispanic, American Indian/Native American, and Other]), and first language spoken at home (English, other). *Parent-reported* covariates were parental education (less than high school, high school, some college, college graduate), parental marital status (married, unmarried [including divorced, separated, widowed, and never married]), and household income (quartiles: $0-$19,999, $20,000-$34,999, $35,000-$49,999, ≥$50,000). Urbanicity of each adolescents’ school location (urban, suburban, rural) was taken from the US Census Quality Education Data (Ingels et al., 1994).

### Statistical analyses

This study aimed to investigate whether arts and cultural engagement in mid-adolescence was associated with RACBs throughout adolescence, and whether attitudes towards RACBs and self-control scores were mediators of this association. Structural equation modelling (SEM) across the three waves of Add Health and NELS:88 allowed the investigation of both the direct and indirect associations between arts and cultural engagement, RACBs, and attitudes/self-control scores whilst accounting for the relationships between them.

**Fig. 1:**
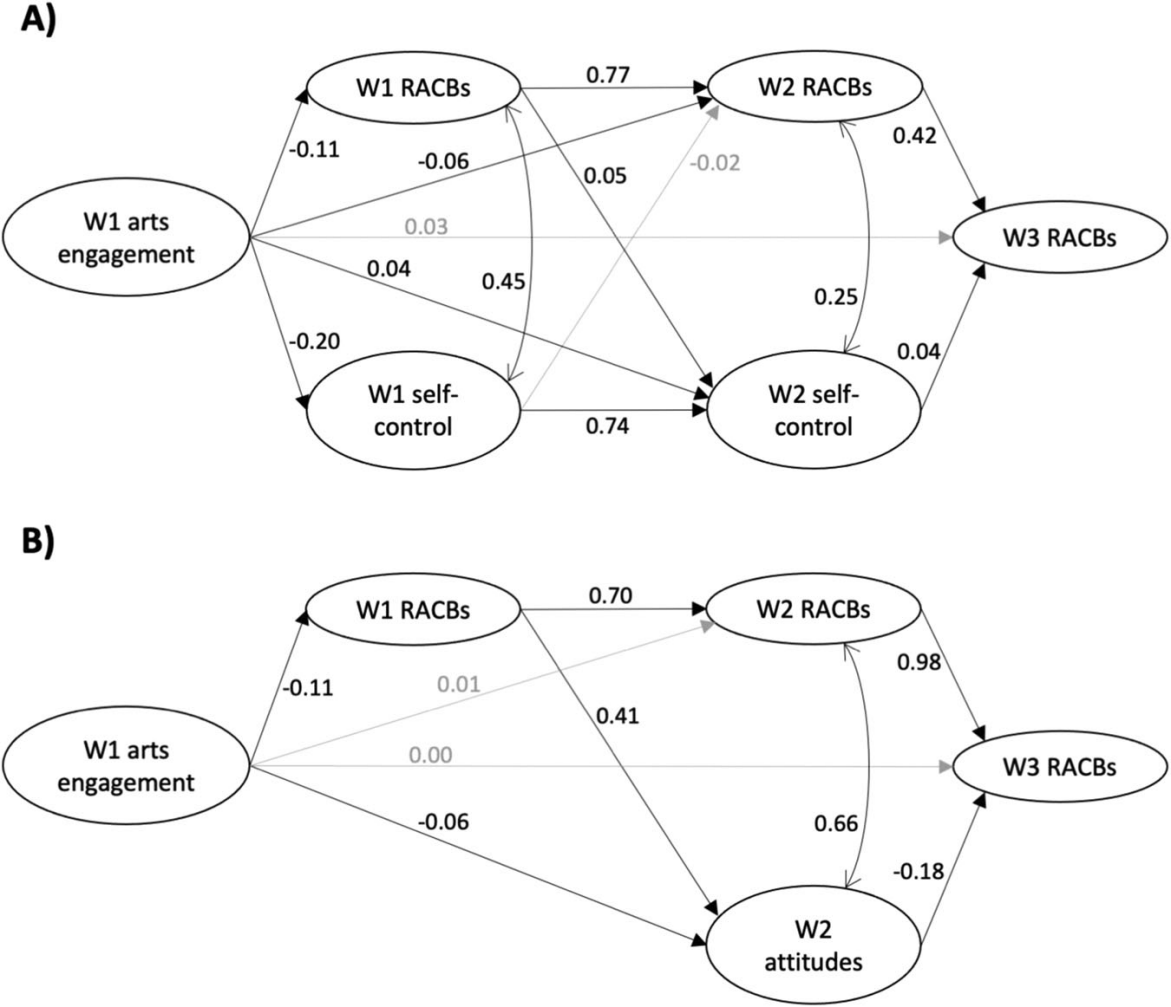
Structural equation models in (**A**) the Add Health Cohort and (**B**) the NELS:88 cohort. Only the structural model and the variables of interest are shown. The prefix indicates the wave at which each latent variable was measured. Standardized coefficients are presented and those in light grey did not reach significance (*p* > 0.05). Standardized coefficients can be interpreted as the change in the outcome (in outcome standard deviation units) for a standard deviation change in the exposure. Arts engagement Arts and cultural engagement. RACBs Reportedly antisocial or criminalized behaviors. Attitudes Attitudes towards reportedly antisocial or criminalized behavior

The SEMs were constructed based on existing literature and logical assumptions about the temporal ordering of covariates. For example, all covariates were modelled as exogenous variables, so could only act as influencers of other factors, and could not be influenced themselves. Socioeconomic position (SEP) was a latent variable indicated by parental education, parental marital status, and household income. Covariates (age, gender, race/ethnicity, first language, urbanicity, SEP) at wave one were allowed to influence arts and cultural engagement at wave one, attitudes/self-control scores at waves one to two, and RACBs at waves one to three (Figs. S1, S2). SEMs modelled the influence of arts and cultural engagement on concurrent and subsequent behavior and attitudes/self-control scores, as well as allowing behavior and attitudes/self-control scores to predict behavior and attitudes/self-control scores at the subsequent wave (Fig. 1). Behavior and attitudes/self-control scores within the same wave were also allowed to correlate with each other. Factor loadings and correlations were allowed to vary across waves as constraining them worsened model fit in both cohorts, indicating that RACBs and attitudes/self-control scores changed developmentally.

Descriptive analyses were performed in Stata 16 (StataCorp, 2019) and the SEMs were fitted in Mplus 8 (Muthén & Muthén, 2017). The SEMs were estimated using a robust weighted least squares estimator with a diagonal weight matrix that is designed for categorical variables (WLSMV; Muthén & Muthén, 2017). This approach uses pairwise deletion for indicator variables and drops participants with missing data on exogenous covariates (Add Health *n* = 216; NELS:88 *n* = 338). If data are missing at random, this approach approximates maximum likelihood estimation. Standard errors and indirect effects were computed using the delta method. As participants were clustered within schools, school was included as a higher-level variable in the SEM and robust standard errors calculated. All other variables were included at the individual-level. Analyses were weighted using the individual-level panel (wave one to wave three) weights from Add Health and NELS:88, making the samples representative of the target adolescent population in the US (1994–1995 grades 7–11 in Add Health, 1988 grade 8 in NELS:88).

The mean- and variance-adjusted chi-square statistic are reported alongside other model fit indices that are less sensitive to sample size, including the root mean square error of approximation (RMSEA), standardized root mean square residual (SRMR), comparative fit index (CFI), and the Tucker-Lewis index (TLI). Coefficients and 95% confidence intervals from the SEM were all standardized using the variances of the continuous latent variables as well as the variances of the background and outcome variables. Standardized coefficients can thus be interpreted as the change in the outcome (in outcome standard deviation units) for a standard deviation change in the exposure.

#### Sensitivity analyses

Sensitivity analyses first explored whether there were gender differences in the associations between arts and cultural engagement and RACBs. This was done by modelling the fully adjusted SEM separately according to gender (male, female) in both Add Health and NELS:88, testing whether similar significant paths hold in gender-specific models. To do this in Add Health, categories of some items on the RACB scale had to be collapsed due to low frequencies (Table S1).

Second, sensitivity analyses explored whether arts and cultural engagement was associated specifically with violent RACBs. To do this, the six questions about violent RACBs in the past 12 months from the Add Health RACBs measure were used (seriously injure someone, use or threaten someone with a weapon, take part in group fight, use a weapon in a fight, pull a knife or gun on someone, shoot or stab someone). The violent RACBs subscale had good to acceptable internal consistency across waves (wave 1 α = 0.71, wave 2 α = 0.74, wave 3 α = 0.62).

## Results

### Add Health Cohort

#### Sample characteristics

After weighting of the 10,610 participants, 50% identified as female and 72% identified as White (Table 1). The mean age was 15.07 years (standard error [SE] = 0.02, range 11–21) at wave one, 15.95 (SE = 0.02, range 12–21) at wave two, and 21.40 (SE = 0.02, range 18–27) at wave three. Adolescents had engaged in an average of 1.69 (SE = 0.02) arts and cultural activities (range = 0–10).

**Table 1:** Descriptive statistics in the first wave in both cohorts

	Add Health (*n* = 10,106)	NELS:88 (*n* = 15,214)
	Mean/ Proportion	Proportion
Age (years)	15.07	-
Age (groups)		
≤13 years	-	1%
14 years	-	63%
15 years	-	31%
≥16 years	-	5%
Gender		
Male	50%	50%
Female	50%	50%
Race/ethnicity		
White	72%	73%
Black	16%	13%
Asian/Pacific Islander	4%	3%
Other	8%	11%
First language		
English	93%	91%
Other	7%	9%
Urbanicity		
Urban	33%	25%
Suburban	28%	44%
Rural	29%	31%
Parental education		
Less than high school	11%	10%
High school	32%	20%
Some college	21%	42%
College graduate	35%	28%
Parental marital status		
Married	73%	80%
Unmarried	27%	20%
Household income (quartiles)		
1	24%	27%
2	26%	29%
3	29%	21%
4	21%	23%

Results weighted. In Add Health, some participants were missing data on parental education (*n* = 504; 5%), parental marital status (*n* = 697; 7%), and household income (*n* = 1819, 18%). In NELS:88, some participants were missing data on parental education (*n* = 6; 0.004%), parental marital status (*n* = 371; 2%), and household income (*n* = 641; 4%). Household income quartiles differed slightly across cohorts (Add Health $0-$20,000, $21,000-$38,000, $39,000-$60,000, $61,000+; NELS:88 $0–$19,999, $20,000–$34,999, $35,000–$49,999, $50,000+)

There were small but significant differences in the characteristics of adolescents who were included and excluded from this study (Table S3); adolescents who completed all waves of Add Health were more likely to be engaged in more arts activities, younger, female, of White race/ethnicity, have English as their first language, live in a rural area, and have married parents with higher levels of education and higher household income than the original Add Health sample. Despite this, weighting adjusted the distribution of the sample accordingly, and there were no differences in RACBs or self-control at wave one between adolescents included in and excluded from this study (Table S3).

The SEM had an acceptable fit to the data ﻿(χ^2^(2,397) = 8594.37, RMSEA = 0.02, SRMR = 0.10, CFI = 0.84, TLI = 0.83). Full results from the model are presented in Fig. S1 and Table S4.

#### Arts engagement and RACBs

More arts and cultural engagement was associated with fewer RACBs at wave one (coefficient [coef] = −0.11, 95% confidence interval [CI] = −0.15 to −0.07; Fig. 1A, Table 2). Overall, more arts and cultural engagement was also associated with fewer RACBs at wave two (total effect coef = −0.14, 95% CI = −0.18 to −0.10). This was driven by a direct association between arts and cultural engagement and RACBs at wave two (coef = −0.06, 95% CI = −0.10 to −0.05), as well as an indirect effect through RACBs at wave one (coef = −0.08, 95% CI = −0.12 to −0.05). Finally, although arts and cultural engagement was not directly associated with RACBs at wave three, it did predict RACBs at wave three indirectly (total indirect coef = −0.06, 95% CI = −0.08 to −0.04), with a significant path through RACBs at waves one and two (coef = −0.03, 95% CI = −0.05 to −0.02). However, after accounting for the other paths in the model, the total effect of arts and cultural engagement on RACBs at wave three was not significant.

**Table 2:** Standardized effects of interest from the full structural equation models (SEMs) in both cohorts

	Coef.	95% CI	*p* value
**Add Health**			
ARTS & CULTURAL ENGAGEMENT → WAVE 1 SELF-CONTROL			
Direct: arts – W1 self-control	**−0.20**	**−0.24 to −0.17**	**<0.001**
ARTS & CULTURAL ENGAGEMENT → WAVE 1 BEHAVIOR			
Direct: arts – W1 behavior	**−0.11**	**−0.15 to −0.07**	**<0.001**
ARTS & CULTURAL ENGAGEMENT → WAVE 2 SELF-CONTROL			
Total	**−0.12**	**−0.15 to −0.08**	**<0.001**
Total indirect	**−0.16**	**−0.18 to −0.13**	**<0.001**
Indirect: arts – W1 self-control – W2 self-control	**−0.15**	**−0.18 to −0.12**	**<0.001**
Indirect: arts – W1 behavior – W2 self-control	**−0.01**	**−0.01 to −0.001**	**0.029**
Direct: arts – W2 self-control	**0.04**	**0.01 to 0.07**	**0.024**
ARTS & CULTURAL ENGAGEMENT → WAVE 2 BEHAVIOR			
Total	**−0.14**	**−0.18 to −0.10**	**<0.001**
Total indirect	**−0.08**	**−0.11 to −0.05**	**<0.001**
Indirect: arts – W1 self-control – W2 behavior	0.01	−0.002 to 0.01	0.243
Indirect: arts – W1 behavior – W2 behavior	**−0.08**	**−0.12 to −0.05**	**<0.001**
Direct: arts – W2 behavior	**−0.06**	**−0.10 to −0.05**	**0.007**
ARTS & CULTURAL ENGAGEMENT → WAVE 3 BEHAVIOR			
Total	**−**0.03	**−**0.08 to 0.02	0.287
Total indirect	**−0.06**	**−0.08 to −0.04**	**<0.001**
Indirect: arts – W2 self-control – W3 behavior	0.002	0.00 to 0.003	0.113
Indirect: arts – W2 behavior – W3 behavior	**−0.03**	**−0.04 to −0.01**	**0.009**
Indirect: arts – W1 self-control – W2 self-control – W3 behavior	**−0.01**	**−0.01 to −0.001**	**0.037**
Indirect: arts – W1 behavior – W2 self-control – W3 behavior	0.00	0.00 to 0.00	0.117
Indirect: arts – W1 self-control – W2 behavior – W3 behavior	0.002	−0.001 to 0.01	0.248
Indirect: arts – W1 behavior – W2 behavior – W3 behavior	**−0.03**	**−0.05 to −0.02**	**<0.001**
Direct: arts – W3 behavior	0.03	**−**0.01 to 0.08	0.244
**NELS:88**			
ARTS & CULTURAL ENGAGEMENT → WAVE 1 BEHAVIOR			
Direct: arts – W1 behavior	**−0.11**	**−0.16 to −0.07**	**<0.001**
ARTS & CULTURAL ENGAGEMENT → WAVE 2 ATTITUDES			
Total	**−0.10**	**−0.14 to −0.07**	**<0.001**
Total indirect	**−0.05**	**−0.06 to −0.03**	**<0.001**
Indirect: arts – W1 behavior – W2 attitudes	**−0.05**	**−0.06 to −0.03**	**<0.001**
Direct: arts – W2 attitudes	**−0.06**	**−0.10 to −0.02**	**0.015**
ARTS & CULTURAL ENGAGEMENT → WAVE 2 BEHAVIOR			
Total	**−0.07**	**−0.11 to −0.03**	**0.003**
Total indirect	**−0.08**	**−0.11 to −0.05**	**<0.001**
Indirect: arts – W1 behavior – W2 behavior	**−0.08**	**−0.11 to −0.05**	**<0.001**
Direct: arts – W2 behavior	0.01	**−**0.04 to 0.05	0.814
ARTS & CULTURAL ENGAGEMENT → WAVE 3 BEHAVIOR			
Total	**−**0.05	**−**0.10 to **−**0.01	0.054
Total indirect	**−0.05**	**−0.09 to −0.02**	**0.020**
Indirect: arts – W2 attitudes – W3 behavior	**0.01**	**0.00 to 0.01**	**0.029**
Indirect: arts – W2 behavior – W3 behavior	0.01	**−**0.04 to 0.05	0.814
Indirect: arts – W1 behavior – W2 attitudes – W3 behavior	**0.01**	**0.00 to 0.01**	**0.002**
Indirect: arts – W1 behavior – W2 behavior – W3 behavior	**−0.08**	**−0.11 to −0.05**	**<0.001**
Direct: arts – W3 behavior	0.00	**−**0.05 to 0.05	0.953

The prefix indicates the wave at which each latent variable was measured. Bold text indicates *p* < 0.05. Standardized coefficients can be interpreted as the change in the outcome (in outcome standard deviation units) for a standard deviation change in the exposure

#### Arts engagement and self-control

More arts and cultural engagement was associated with better concurrent self-control scores at wave one (coef = −0.20, 95% CI = −0.24 to −0.17). There was also some weak evidence that more arts and cultural engagement was directly associated with worse self-control scores at wave two (coef = 0.04, 95% CI = 0.01 to 0.07), but the total effect of arts and cultural engagement on self-control scores at wave two was in the opposite direction (coef = −0.12, 95% CI = −0.15 to −0.08). This was driven by paths through other factors (total indirect coef = −0.16, 95% CI = −0.18 to −0.13), such that more engagement was associated with better self-control scores at wave two through self-control scores at wave one (coef = −0.15, 95% CI = −0.18 to −0.12) and RACBs at wave one (coef = −0.01, 95% CI = −0.01 to −0.001).

#### RACBS and self-control

Worse self-control was associated with more RACBs concurrently at both wave one (coef = 0.45, 95% CI = 0.42 to 0.47) and wave two (coef = 0.25, 95% CI = 0.20 to 0.30).

#### Mediation by self-control

There was no evidence that the association between arts and cultural engagement and RACBs at wave two was driven by an effect through self-control scores at wave one. There was weak evidence for a significant path from arts and cultural engagement to RACBs at wave three through self-control scores at waves one and two (coef = −0.01, 95% CI = −0.01 to −0.001).

#### Effects of covariates

Lower socioeconomic position (SEP) was associated with lower arts and cultural engagement (Table S4), more RACBs at wave one (but fewer RACBs at wave three), and worse self-control scores at wave one. Compared to younger adolescents at wave one, older participants had less arts and cultural engagement, fewer RACBs at wave two and three, and worse self-control scores at wave one (but better self-control scores at wave two). Gender was not associated with arts and cultural engagement, but females did have fewer RACBs at waves one and three and worse self-control at wave one than males. Adolescents of Black race/ethnicity reported lower arts and cultural engagement, more RACBs at wave one and three, and better self-control at wave one than White participants. Other racial/ethnic groups did not differ to White participants, except that those of Asian/Pacific Islander race/ethnicity had worse self-control scores at wave two. Finally, urbanicity was associated with RACBs, as adolescents living in rural areas reported fewer RACBs at wave one than those living in urban areas.

### NELS:88 Cohort

#### Sample characteristics

After weighting of the 15,214 participants, 50% identified as female and 73% identified as White (Table 1). At wave one, 63% of participants were 14 and 31% were 15 years old. Most participants were aged 15-16 years at wave two and 17–18 years at wave three. Adolescents had engaged in an average of 5.06 (SE = 0.03) arts and cultural activities (range = 0–14).

There were small but significant differences in the characteristics of adolescents who were included and excluded from this study (Table S3); adolescents who completed all waves of NELS:88 reported fewer RACBs and were more likely to be engaged in more arts activities, younger, of White race/ethnicity, have English as their first language, live in a rural area, and have married parents with higher levels of education and higher household income than the original NELS:88 sample. Weighting for attrition adjusted the distribution of the sample accordingly.

The full SEM had an acceptable fit to the data ﻿(χ^2^(1,076) = 5047.56, RMSEA = 0.02, SRMR = 0.07, CFI = 0.92, TLI = 0.91). The full results from the model are presented in Fig. S2 and Table S5.

#### Arts engagement and RACBs

More arts and cultural engagement was associated with fewer RACBs concurrently at wave one (coef = −0.11, 95% CI = −0.16 to −0.07; Fig. 1B, Table 2). Overall, more engagement was also associated with fewer RACBs at wave two (total effect coef = −0.07, 95% CI = −0.11 to −0.03). Although arts and cultural engagement was not directly associated with RACBs at wave two, this was driven by an indirect effect through RACBs at wave one (coef = −0.08, 95% CI = −0.11 to −0.05). Finally, although not directly associated, there was weak evidence that more engagement was associated with fewer RACBs overall at wave three (total effect coef = −0.05, 95% CI = −0.10 to −0.01). This association was the result of several significant indirect effects, including through RACBs at waves one and two (coef = −0.08, 95% CI = −0.11 to −0.05).

#### Arts engagement and attitudes towards RACBs

Overall, more arts and cultural engagement was also associated with fewer positive perceptions of RACBs at wave two (total effect coef = −0.10, 95% CI = −0.14 to −0.07). In addition to the direct association between arts and cultural engagement and attitudes at wave two (coef = −0.06, 95% CI = −0.10 to −0.02), there was also evidence for an indirect association through RACBs at wave one (coef = −0.05, 95% CI = −0.06 to −0.03).

#### RACBs and attitudes towards RACBs

More positive perceptions of RACBs at wave two were strongly associated with more RACBs at wave two (coef = 0.66, 95% CI = 0.62 to 0.69), but fewer RACBs at wave three (coef = −0.18, 95% CI = −0.25 to −0.12). This negative longitudinal association was likely due to the very strong association between RACBs at wave two and three (coef = 0.98, 95% CI = 0.91–1.05).

#### Mediation by attitudes towards RACBs

There was some evidence that attitudes towards RACBs mediated the overall association between arts and cultural engagement and RACBs at wave three, but this was in the opposite direction to that expected. There were very small but significant paths from more arts and cultural engagement at wave one to more RACBs at wave three through: attitudes at wave two (coef = 0.01, 95% CI = 0.00 to 0.01); and both RACBs at wave one and attitudes at wave two (coef = 0.01, 95% CI = 0.00 to 0.01).

#### Effects of covariates

Higher SEP was associated with more arts and cultural engagement and fewer RACBs at wave one, but not with attitudes towards RACBs at wave two. Compared to the youngest adolescents (≤13 years), the oldest participants (≥16 years) had lower arts and cultural engagement and adolescents aged 14 had more RACBs at wave two, but there were no other associations with age. Females had higher arts and cultural engagement, fewer RACBs at all waves, and fewer positive perceptions of RACBs. Compared to White participants, Black adolescents and those of Other race/ethnicity (including Hispanic, American Indian/Native American, and Other) had lower arts and cultural engagement, more RACBs at wave one, and fewer positive perceptions of RACBs at wave two. Adolescents of Asian/Pacific Islander race/ethnicity had more arts and cultural engagement and fewer RACBs at waves one and two than White participants. Finally, urbanicity was only associated with arts and cultural engagement, as adolescents living in rural areas reported lower engagement than those living in urban areas.

### Sensitivity Analysis: Gender

#### Add health

In Add Health, the SEM modelled separately according to gender had an acceptable fit to the data (χ^2^(4,149) = 10,538.33, RMSEA = 0.02, SRMR = 0.09, CFI = 0.84, TLI = 0.84). In this sample, there were few gender differences in the associations between arts and cultural engagement, self-control, and RACBs (Table S6). As in the main analyses, across both males and females, more arts and cultural engagement was associated with fewer RACBs at wave one. Similarly, in both genders, more arts and cultural engagement was indirectly associated with fewer RACBs at waves two and three. Although arts and cultural engagement was directly associated with more RACBs at wave three in females (but not males), the indirect effects through previous RACBs and self-control led to fewer RACBs at wave three overall. In males, the association with fewer RACBs at wave three occurred only through previous RACBs. There was thus some weak evidence that self-control mediated the association between arts and cultural engagement and RACBs at wave three for females, and not for males. However, more arts and cultural engagement was associated with better self-control scores at wave one, and wave two indirectly through self-control at wave one, in both genders. For males, more arts and cultural engagement was also directly associated with worse self-control at wave two. Across most of these paths, the associations between arts and cultural engagement, self-control, and RACBs were stronger in females than males.

#### NELS:88

In NELS:88, the SEM modelled separately according to gender had an acceptable fit to the data (χ^2^(2,147) = 6,767.40, RMSEA = 0.02, SRMR = 0.10, CFI = 0.92, TLI = 0.91). In this sample, as in the main analyses, more arts and cultural engagement was associated with fewer RACBs at waves one and two in both genders. The association at wave two was primarily a result of the indirect path through RACBs at wave one, although this effect was larger in males than females. Overall, arts and cultural engagement was only associated with RACBs at wave three in males, which was mainly due to the indirect path through previous RACBs. Although more arts and cultural engagement was associated with fewer positive perceptions of RACBs in both genders, this association occurred through different paths. In males, the association was driven by an indirect path through RACBs at wave one. In females, the association was driven by the direct path from arts and cultural engagement to attitudes at wave two. There was some evidence that attitudes mediated the association between arts and cultural engagement and RACBs at wave three in both genders, but these were very small effects.

### Sensitivity Analysis: Violent RACBs

A second sensitivity analysis explored whether arts and cultural engagement was associated specifically with violent RACBs in Add Health. This SEM had an acceptable fit to the data (χ^2^(1,452) = 7501.47, RMSEA = 0.02, SRMR = 0.10, CFI = 0.82, TLI = 0.81). The results from this analysis were very similar to the main analyses (Table S7). Two indirect paths no longer reached significance: a) from arts and cultural engagement to self-control at wave two through violent RACBs at wave one, and b) from arts and cultural engagement to violent RACBs at wave three through self-control at waves one and two. Although there was also some evidence that arts and cultural engagement led to more violent RACBs at wave three, the overall association between these variables was in the opposite direction. Findings were thus generally replicated when considering violent RACBs alone, although self-control is less likely to mediate the association between arts and cultural engagement and violent RACBs.

## Discussion

Arts and cultural engagement is a potential strategy for reducing or preventing reportedly antisocial or criminalized behaviors in adolescence. However, most research to date has focused on arts-based interventions and has not tested arts and cultural engagement in large population-based longitudinal studies. There are a range of potential mechanisms through which arts and cultural engagement may reduce reportedly antisocial or criminalized behaviors, from which attitudes towards these behaviors and self-control have been identified as key factors (Social Exclusion Unit, 2002). Yet, it is currently unclear whether ubiquitous arts and cultural engagement, which is not a targeted intervention, can lead to changes in attitudes and self-control. Research to date has not tested whether these mechanisms mediate the association between arts and cultural engagement and behavior. This study therefore aimed to investigate whether overall arts and cultural engagement in mid-adolescence influenced reportedly antisocial or criminalized behaviors in mid- to late adolescence, and also aimed to test two distinct potential mechanisms linking these behaviors: attitudes towards reportedly antisocial or criminalized behaviors and self-control. Across two large longitudinal studies in the US, there was evidence that more arts and cultural engagement was associated with reduced reportedly antisocial or criminalized behaviors concurrently and one to two years later, as well as some limited evidence that this association could be mediated by both attitudes and self-control.

Across both Add Health and NELS:88, more arts and cultural engagement was concurrently associated with fewer reportedly antisocial or criminalized behaviors. Associations were sustained one to two years later, mainly through earlier reportedly antisocial or criminalized behaviors, except for a direct association between arts and cultural engagement and behavior one year later in Add Health. In terms of longer follow-ups, in NELS:88, this relationship was maintained four years later, but only indirectly through earlier behaviors. Similarly, in Add Health, this relationship was only maintained seven years later because of earlier behavior. This is not surprising given that prior occurrence of reportedly antisocial or criminalized behavior is a strong predictor of future behavior in adolescence (Perez et al., 2018). Although this study hypothesized that arts and cultural engagement would reduce subsequent behavior, it is possible that this association is due to reverse causality. Adolescents with more reportedly antisocial or criminalized behavior may be less likely to engage in the arts, giving rise to the associations found here. The direct association between arts and cultural engagement and behavior one year later in Add Health indicates that this is not the case; engagement was associated with subsequent behavior even after adjusting for previous behavior. Overall, these findings provide preliminary evidence that arts and cultural engagement may reduce subsequent reportedly antisocial or criminalized behavior. This adds to the limited existing evidence that participation in performing and fine arts is associated with lower rates of skipping school (Eccles & Barber, 1999), dropping out of school (McNeal, 1995), and being arrested (Zill et al., 1995) and contrasts with research finding no evidence for this association (Fauth et al., 2007).

In this study, results were remarkably consistent across the two cohorts, despite differences in participant age, time scale, and measures of arts and cultural engagement and reportedly antisocial or criminalized behaviors. Add Health followed individuals from early adolescence into young adulthood, whereas NELS:88 focused on the teen years. This indicates that the potential benefits of arts and cultural engagement are not limited to childhood or early adolescence but may occur across this whole developmental period. The differences in the measures included in each cohort should also be considered when interpreting these findings. As done previously (Martin et al., 2013), this study aimed to measure overall engagement in arts and culture as an overarching construct, and thus maximized the number of items measuring engagement in each cohort. This meant that Add Health mainly included items related to participation in school arts clubs, although questions on family attendance at cultural events also loaded highly onto the latent factor. In contrast, NELS:88 included a broader measure of arts and cultural engagement, which had higher internal consistency and was most strongly determined by parent reports of attendance at arts and cultural classes, venues, and events. However, both latent variables still indicated overall levels of engagement in a range arts and cultural activities (e.g., participatory, receptive, within school, outside school) so, despite some differences, it is not surprising that both forms of arts and cultural engagement were associated with lower reportedly antisocial or criminalized behaviors. Perhaps more different across cohorts were the measures of reportedly antisocial or criminalized behaviors. NELS:88 focused on a narrower range of school-based reportedly antisocial behaviors, whereas Add Health measured a range of criminalized behaviors occurring mainly outside of school, all of which may also be considered antisocial. As there is currently a lack of population-level evidence on arts and cultural engagement and these behaviors, this study aimed to provide preliminary evidence on whether engagement could reduce or prevent a wide range of behaviors. The findings demonstrate that arts and cultural engagement has potential for reducing various types of behavior, all of which could be investigated in more detail in future research. In summary, the replication of findings across two cohorts that are not directly comparable suggests that the results are conceptually robust and relevant from mid-adolescence to young adulthood.

### Potential Mechanisms

To extend previous findings, this study sought to identify mediating factors that could explain the relationship between arts and cultural engagement and reportedly antisocial or criminalized behaviors. Two distinct potential mediators were tested: self-control and attitudes towards reportedly antisocial or criminalized behaviors. In Add Health, more arts and cultural engagement was associated with higher self-control scores concurrently and one to two years later. This is in line with previous evidence that self-control is improved by participating in arts programs in the legal system (Bilby et al., 2013), national orchestras (Alemán et al., 2017), singing (Moon, 2017), dance programs (Milliken, 2002), and theater-based interventions (Farhadi & Tabatabaei Zavareh, 2020). Worse self-control was consistently associated with more reportedly antisocial or criminalized behaviors, also consistent with previous evidence (Wolfe & Hoffmann, 2016). Despite this, there was only weak evidence that self-control mediated the association between arts and cultural engagement and behavior at wave three, and this was inconsistent, as self-control did not mediate the association with behavior at wave two. This could be because the association between arts and cultural engagement and self-control is due to reverse causality, or because self-control is a relatively stable trait by adolescence (Britt & Gottfredson, 2011), which is not modified by arts and cultural engagement. In a previous randomized trial, a music program improved self-control in participants aged up to 14 years (Alemán et al., 2017), providing evidence against both possibilities. Future research should therefore explore the developmental changes in associations between arts and cultural engagement, self-control, and reportedly antisocial or criminalized behaviors. Overall, even if self-control does not mediate the association with behaviors, an effect of arts and cultural engagement on self-control may still benefit a range of other outcomes, such as social functioning (Gottfredson & Hirschi, 1990), emotion regulation, wellbeing (Wenzel et al., 2021), and other health behaviors (Boisvert et al., 2013).

In NELS:88, more arts and cultural engagement was longitudinally associated with fewer positive perceptions of reportedly antisocial or criminalized behaviors. This is consistent with previous evidence that young people’s attitudes may be improved by arts-based interventions in the legal system (Hughes, 2005) and community music sessions (Clennon, 2013). Fewer positive perceptions were also associated with fewer reportedly antisocial or criminalized behaviors concurrently, but the direction of this association was reversed longitudinally. Therefore, although there was evidence for attitudes as a mediator, this was in the opposite direction to that hypothesized: arts and cultural engagement led to fewer positive perceptions of reportedly antisocial or criminalized behaviors, which then led to more behaviors. However, this evidence was weak, and the coefficient was very small. This finding could be because of the questions included in NELS:88, which measured *general attitudes* towards reportedly antisocial or criminalized behaviors but measured only *school-based behaviors*. Regardless of this, attitudes are an important intermediate outcome in and of themselves (Clawson & Coolbaugh, 2001), and are associated with spending more time with peers with similar attitudes (Brendgen et al., 2000), decreased wellbeing (Phillips & Pittman, 2007), and lower academic achievement (Chang & Le, 2005). It is thus promising that ubiquitous arts and cultural engagement may reduce positive perceptions of reportedly antisocial or criminalized behaviors.

Despite previous evidence for gender differences in the associations between extracurricular participation, reportedly antisocial or criminalized behaviors (Linville & Huebner, 2005), and school misconduct (Miller et al., 2005), the findings in this study were mostly replicated across genders. More arts and cultural engagement was associated with fewer behaviors up to seven years later in males and females across both cohorts. The short-term associations, with behavior up to one year later, appeared slightly larger in females. This could indicate that arts and cultural engagement is more beneficial for females, which is supported by evidence that self-control only mediated this association in females, and not males. Given that this was an exploratory sensitivity analysis, future research should investigate this possibility further. In a second sensitivity analysis, the findings of this study were replicated when including only violent behaviors, such as using a weapon, fighting, and seriously injuring someone. Violence is more likely to be perceived as antisocial or a criminalized behavior across cultures, so may be a less subjective outcome. The replication of study findings (except for a lack of mediation by self-control) specifically for violent behaviors demonstrates the critical policy relevance of promoting arts and cultural engagement for the prevention of these behaviors in adolescence.

More research is needed to explore other potential moderators and mediators of the relationship between arts and cultural engagement and reportedly antisocial or criminalized behaviors. When considering potential mediators, a review found that arts and cultural engagement is associated with increased empathy and prosocial behavior (Konrath & Kisida, 2021). Another systematic review found evidence that lower empathy is associated with more offending (Jolliffe & Farrington, 2004). Similarly, reductions in prosocial behavior throughout adolescence are associated with increases in aggression and “delinquency” (Padilla-Walker et al., 2018). In addition, arts and cultural engagement may enhance emotion regulation (Fancourt & Ali, 2019) and self-esteem (Mak & Fancourt, 2019), and improvements in both emotion regulation (Rodriguez et al., 2016) and self-esteem (Donnellan et al., 2005) may reduce reportedly antisocial or criminalized behaviors. These activities may also allow safe exploration of boundaries in expression, providing opportunities for learning from risk-taking. Furthermore, many arts and cultural activities are structured in nature, as they involve adult supervision, rule-guided engagement, skill development, sustained attention, and regular schedules (Mahoney, 2000). This may be beneficial, as there is evidence that structured extracurricular activities reduce reportedly antisocial or criminalized behaviors, in contrast to unstructured activities (Mahoney & Stattin, 2000). Future studies should seek to understand whether both structured and unstructured arts and cultural engagement reduce reportedly antisocial or criminalized behaviors while also accounting for the role of other structured activities in adolescence. Additionally, in this study, the broad definition of arts and cultural engagement included both participatory engagement in artistic and creative activities, as well as receptive engagement such as visiting museums and attending performances (Fancourt & Finn, 2019). Further research could investigate whether participatory and receptive engagement have differential effects on reportedly antisocial or criminalized behaviors in adolescence.

There is also some evidence that the positive impact of extracurricular activities on reportedly antisocial or criminalized behaviors relies on adolescents’ peers being engaged in similar activities (Mahoney, 2014). Adolescence is an important developmental period of social reorientation, in which individuals become more susceptible to peer influence and more sensitive to peer rejection (Andrews et al., 2020). Although measures of peer reportedly antisocial or criminalized behaviors or peer arts and cultural engagement could not be included in this study, arts and cultural activities can provide a positive environment in which adolescents are likely to be involved with a peer group who may encourage healthy behaviors and lifestyle choices. Future research should investigate the role of adolescents’ peer networks in the relationship between arts and cultural engagement and reportedly antisocial or criminalized behaviors.

### Implications and Challenges in “Delinquency” Research

The findings of this study indicate that arts and cultural engagement could have both short-term and enduring effects on adolescents’ lives, providing opportunities to realize positive developmental outcomes. This demonstrates the need for further research on the role of arts and cultural engagement in supporting health-promoting behaviors in adolescence. In addition to advancing related research, the next priority should be to ensure different forms of arts and cultural engagement can be made accessible, socially inclusive, and culturally appropriate for all young people. This is particularly important given that, when adjusted for inflation, funding for the arts in schools has decreased by 30% over the past twenty years in the US (Jung, 2018). Additionally, the US government has repeatedly proposed cutting all federal arts and cultural funding, and there are frequent debates about the extent to which the arts should be part of school curricula (e.g., McGlone, 2020). Given the time that children spend in school, as well as barriers to and increased social gradients in participation in arts and cultural activities outside of school (Mak & Fancourt, 2021), this study’s findings underscore the importance of curricular and extracurricular arts programs at schools and the need for policies that ensure funding for arts in education. These findings also support the use of the arts in rehabilitation programs for adolescents in the legal system, both for males and females, and for those with a history of non-violent and violent behavior. Additionally, work is currently underway in other countries such as the UK as well as in pilots in the US to bring arts to adolescents via social prescribing (SP) schemes. SP usually involves a health, social or educational professional referring an adolescent to a link worker, who develops a plan that connects the adolescent with psychosocial activities such as the arts with the aim of improving psychological or social wellbeing. Preliminary studies involving adolescents have shown benefits for mental health, the development of social networks, reductions in loneliness, and reductions in feelings of stigma (Bertotti et al., 2020). Consequently, SP schemes could be explored further for adolescents considered at risk for developing reportedly antisocial or criminalized behaviors.

Language is a significant issue in this area of research. Throughout this study, the term “reportedly antisocial or criminalized behaviors” has been used as an alternative to “delinquent behaviors”. “Reportedly antisocial” highlights that pro- and antisociality are assigned rather than inherent types of behavior. “Criminalized” highlights that behavior does not have inherent legality or illegality but that certain behaviors and circumstances have been criminalized, including for particular groups (such as alcohol consumption by under-18s). This characterization may or may not accurately and adequately reflect an adolescent’s perception of their own behavior. Although the current literature regularly uses the term “delinquency”, this term is problematic, as it contributes to the association of criminality with behaviors that often result from adversity. The significance of early life adversity and maltreatment to health, development, and life course trajectories is well-established (Struck et al., 2021). Adverse childhood experiences (ACEs) include abuse, neglect, and household dysfunction, and over 60% of Americans have experienced at least one ACE (CDC, 2021). Young people with these experiences are often at greater risk of reportedly antisocial or criminalized behaviors and becoming involved in the legal system (Baglivio et al., 2015). Terms like “delinquency” also lack consideration of other important factors such as neurobiology (Zijlmans et al., 2021) and neurodiversity (Lollini, 2018). The noun “delinquent” labels and criminalizes individuals themselves. This kind of labelling could be particularly detrimental in school settings, where much development and identity formation take place.

Additionally, assuming that reductions of certain types of adolescent behaviors is an inherent public good is problematic. Adolescent behaviors that have been associated with criminality, pathologies, or antisociality may in some cases be adaptive responses to destructive or oppressive environments. For example, asserting control outside of a home that does not allow normal levels of control or self-determination may be adaptive, as exercising control is an essential developmental behavior (Bandura, 2006). In addition, reportedly antisocial or criminalized behaviors may elicit positive social responses from peers or be related to different biological antecedents or cultural meanings across groups (Chen, 2020). Attitudes towards reportedly antisocial or criminalized behaviors, including the extent to which adolescents consider that behaviors such as belonging to gangs or stealing are acceptable, may be determined by how safe they feel in their home or school environment and experiences with scarcity of food or other necessary goods. The role of these structural and social determinants may be obscured by the criminalization or pathologizing of affected adolescents. Assuming that behaviors are detrimental and prioritizing their reduction can convey apparent concern for adolescents’ health while avoiding systemic responsibility for that health by acting at community, organizational, and policy levels. The prevention or reduction of behaviors that have historically been referred to as “delinquent” should not be presumed to indicate a public health success, particularly without analyses of related social and structural determinants of health.

Furthermore, adolescents’ behaviors cannot be assumed to be accurately interpreted or reported by adults and those with relative power. For example, gender norms and stereotypes have caused girls in the juvenile legal system to be labeled as problematically aggressive due to behaviors that would be considered common among boys (Golden, 2017). Attributions and interpretations of delinquency, illegality, sociality, and even health are subjective, and may not be shared across age, gender, race/ethnicity, cultures, or historical moments. As a result, it is critical that data related to adolescent behaviors be interpreted with regard for the potential effects of biases. Despite previous studies’ failures to consistently navigate critical nuances, their data do provide information regarding adolescent behaviors and associations with health outcomes and may also generate insights into more equitable means of collecting and analyzing data in the future. By considering these issues, and not using the problematic language of “delinquency”, this article contributes to this dual effort. In the future, researchers must a) recognize the need for changes in terminology around “delinquency”; b) acknowledge the fact that behaviors are often labeled and interpreted by others; and c) undertake more research into the effects of social and structural determinants of health on adolescent behaviors and health outcomes.

### Strengths and Limitations

This study has several strengths. Two large nationally representative longitudinal studies were used, and findings were replicated despite differences in the cohorts, indicating that the results are conceptually robust. Add Health and NELS:88 were chosen for their rich array of data on arts and cultural engagement and covariates, meaning that sociodemographic factors that are likely to confound the association between arts and cultural engagement and reportedly antisocial or criminalized behaviors could be included (Feldman Farb & Matjasko, 2012). In analyses, participants were clustered within schools, accounting for the fact that adolescents within schools are more similar to each other than to adolescents at other schools. Additionally, reportedly antisocial or criminalized behaviors were modelled across three waves in each cohort. This is important as these behaviors may change developmentally (Cook et al., 2015).

This study also has some limitations. It was limited by the measures of arts and cultural engagement and reportedly antisocial or criminalized behaviors in Add Health and NELS:88, which were not consistent across waves. More sophisticated approaches such as growth curve models therefore could not be used. The internal consistency of these measures differed across cohorts and waves, which could be a result of developmental changes or indicate that heterogeneous constructs were grouped together. However, given that measures were represented by latent factors in structural equation models (SEMs), Cronbach’s alpha is likely to be an underestimate of the reliability of these measures (Sijtsma, 2009), as it is not derived from the parameters of the factor model (Raykov, 1997). This approach assumes that the observed information reflects unmeasurable constructs and accounts for measurement error in latent variables by simultaneously estimating measurement and structural models (Kline, 2015). Yet, using SEMs assumes linear relations between these factors, which may not be appropriate (e.g., Matjasko et al., 2019). Future research should investigate the developmental trajectories of arts and cultural engagement and reportedly antisocial or criminalized behaviors in more detail. Additionally, the measure of reportedly antisocial or criminalized behaviors in NELS:88 included getting into trouble for behavior at school, which may have been influenced by teachers’ beliefs and behavior, but variation in teacher conduct could not be accounted for in analyses.

Furthermore, Add Health and NELS:88 are relatively old cohorts. While these cohorts included nationally representative samples of the target populations (1994-1995 grades 7-11 in Add Health, 1988 grade 8 in NELS:88), these samples are no longer representative of the current US population (US Census Bureau, 2021). It is likely that the associations observed between arts and cultural engagement and reportedly antisocial or criminalized behaviors are due to enduring psychological and social mechanisms, in which case the age of the data is not important. Nevertheless, it is possible that these associations have been altered by changes in children’s educational and developmental environments. Advances in external factors such as technology, educational styles, and behavioral management techniques may have modified the relationship between arts and cultural engagement and reportedly antisocial or criminalized behaviors. The findings of this study should thus by replicated with more recent data, although replication is challenging due to the lack of current data on arts and cultural engagement in representative cohorts.

Although analyses were adjusted for a wide range of sociodemographic factors, it is possible that other characteristics influence both arts and cultural engagement (Mak & Fancourt, 2021) and reportedly antisocial or criminalized behaviors (Shader, 2000), as there is a social gradient in both behaviors. It remains difficult to disentangle whether the association between arts and cultural engagement and reportedly antisocial or criminalized behaviors is due to self-selection or because engaging in arts and cultural activities reduces those behaviors. Additionally, a biased sample may have been included in both cohorts due to attrition, as participants in this study were more likely to be engaged in arts and cultural activities, younger, White, live in a rural area, and have higher socioeconomic position than the baseline Add Health and NELS:88 samples. Weighting adjusted the distribution of both samples accordingly. Future research should also examine whether these associations are moderated by age, race/ethnicity, and other factors that may increase adolescents’ participation in reportedly antisocial or criminalized behaviors (Feldman & Matjasko, 2005).

This study used an overly simple race/ethnicity variable (White, Black, Asian/Pacific Islander, Other) due to small numbers in non-White groups. This approach conflates experiences across diverse racial/ethnic groups, which might be particularly problematic as these groups may not have equal access to artistic and cultural resources (Bone et al., 2021). Future research should thus use more diverse samples and collect more nuanced data on race/ethnicity, while considering the persistence of structural racism in US communities, schools, and legal systems (Williams, 2012). Additionally, many arts and cultural activities take place within groups that are not well represented in this sample, and these activities may not be included in the narrow definitions of arts and cultural engagement used in Add Health and NELS:88. Finally, the findings are limited in that they rely on survey constructs that, to the authors’ knowledge, were not informed by adolescents at the time of the studies, and thus may not adequately reflect their experiences with arts and culture and with reportedly antisocial or criminalized behaviors. To help address such issues in the future, adolescents themselves should be included and given leadership roles in research related to their lives and behaviors (see Checkoway, 2011).

## Conclusion

Arts and cultural engagement is a potential strategy for reducing or preventing reportedly antisocial or criminalized behaviors in adolescence, which may operate through mechanisms including attitudes towards these behaviors and self-control. However, most research to date has focused on arts-based interventions and has not tested arts and cultural engagement in large population-based longitudinal studies. Therefore, in this study, data from two large longitudinal studies was used to investigate whether arts and cultural engagement influenced reportedly antisocial or criminalized behaviors in adolescence and young adulthood and to test whether these associations were mediated by attitudes towards reportedly antisocial or criminalized behaviors and self-control. This study provides the first evidence that more engagement in a range of arts and cultural activities in mid-adolescence was associated with fewer reportedly antisocial or criminalized behaviors, fewer positive perceptions of these behaviors, and higher self-control scores both concurrently and over at least the subsequent two years. Although there was very little evidence that either attitudes or self-control mediated the association between arts and cultural engagement and behavior, the findings indicate that participating in arts and cultural activities may provide opportunities for adolescents to realize positive developmental outcomes. Given the links between reportedly antisocial or criminalized behaviors and health and wellbeing (Walsh et al., 2013), further research is needed on the role of arts and cultural engagement in supporting health-promoting behaviors in adolescence. In addition to advancing related research, the next priority should be to ensure different forms of arts and cultural engagement can be made accessible, socially inclusive, and culturally appropriate for all young people.

## Supplementary Information

Supplementary Materials
