# Correction to: Arts and Cultural Engagement, Reportedly Antisocial or Criminalized Behaviors, and Potential Mediators in Two Longitudinal Cohorts of Adolescents

**DOI:** 10.1007/s10964-022-01673-7

**Published:** 2022-09-01

**Authors:** Jessica K. Bone, Feifei Bu, Meg E. Fluharty, Elise Paul, Jill K. Sonke, Daisy Fancourt

**Affiliations:** 1 Research Department of Behavioural Science and Health, Institute of Epidemiology & Health Care, University College London, London, UK; 2 Center for Arts in Medicine, University of Florida, Gainesville, Florida US

Correction to: Journal of Youth and Adolescence

10.1007/s10964-022-01591-8

In the original publication of the article, the author had neglected to thank Tasha Golden who had contributed her ideas and several paragraphs to this manuscript. This erratum corrects the omission in the Acknowledgement section.

