# Supplementary material for: Arts and Cultural Engagement, Reportedly Antisocial or Criminalized Behaviors, and Potential Mediators in Two Longitudinal Cohorts of Adolescents
Source: J Youth Adolesc. 2022 Mar 23;51(8):1463–82. doi: 10.1007/s10964-022-01591-8 (PMC8940513; doi:10.1007/s10964-022-01591-8)
Supplement: Supplementary file 1 — Supplementary Materials [file 10964_2022_1591_MOESM1_ESM.docx]

# Supplementary Materials

**Table S1.** *Detailed outline of all measures used as indicators for arts and cultural engagement, reportedly antisocial and criminalized behavior, and self-control latent factors in Add Health.*

| **Measure** | **Responses** | **Wave** | **α** |
| --- | --- | --- | --- |
| **Arts and cultural engagement**  Are you participating/Do you plan to participate in the following clubs, organizations, and teams? (check all that apply)   1. Book club 2. Drama club 3. Band 4. Cheerleading or dance 5. Chorus or choir 6. Orchestra 7. Student newspaper 8. During the past week, how many times did you do hobbies?  (Recoded so that 0 = never, 1 = 1+ times) 9. Went to a movie/play/museum/concert/sports event with resident or biological mother in the past four weeks 10. Went to a movie/play/museum/concert/sports event with resident or biological father in the past four weeks | 0 no  1 yes | 1 | 0.41 |
| **Reportedly antisocial and criminalized behavior (RACB)**  In the past 12 months, how often did you:   1. Damage property 2. Steal something worth more than $50* 3. Burglarize a building* 4. Sell drugs 5. Steal something worth less than $50 6. Seriously injure someone 7. Use or threaten someone with a weapon* 8. Take part in group fight* 9. Use a weapon in a fight* 10. Pulled a knife or gun on someone* 11. Shot or stabbed someone* | 0 never  1 1-2 times  2 3-4 times  3 5+ times | 1-3 | 1: 0.78  2: 0.78  3: 0.69 |
| **Self-control**   1. Do you have trouble getting along with your teachers? 2. Do you have trouble paying attention in school? 3. Do you have trouble getting our homework done? 4. Do you have trouble getting along with other students? 5. Do you have trouble keeping your mind focused? 6. When you get what you want, it is usually because you worked hard for it. 7. You like yourself just the way you are. 8. You feel like you are doing everything just about right. 9. You feel socially accepted. | 0 never  1 a few times  2 once a week  3 almost everyday  4 everyday  0 strongly agree  1 agree  2 neither  3 disagree  4 strongly disagree | 1-2 | 1: 0.72  2: 0.72 |

*Note.* A * indicates that the categories of the item were collapsed in the sensitivity analysis testing whether gender was a moderator.

**Table S2.** *Detailed outline of all measures used as indicators for arts and cultural engagement, reportedly antisocial and criminalized behavior (RACB), and attitudes towards RACB latent factors in NELS:88.*

| **Measure** | **Responses** | **Wave** | **α** |
| --- | --- | --- | --- |
| **Arts and cultural engagement**  Have you or will you have participated in any of the following school activities during the current school year, either as a member, or as an officer (for example, vice-president, coordinator, team captain)? (mark all that apply)   1. Band or orchestra 2. Chorus or choir 3. Dance 4. Drama club 5. Student newspaper   Have you or will you have participated in any of the following outside-school activities this year, either as a member, or as an officer (for example, vice-president,  coordinator, team captain)? (mark all that apply)   1. Hobby clubs 2. How much additional reading do you do each week on your own outside school - not in connection with schoolwork? Do not count any reading done for any school purpose. (recoded as a reading indicator variable so that none is no, and anything more than none is yes)   Parent-reported - Has your eighth grader attended classes outside of his or her regular school to study any of the following? (mark all that apply)   1. Art 2. Music 3. Dance 4. The history and culture of his/her ancestors   Parent-reported - Does your eighth grader:   1. Borrow books from the public library? 2. Attend concerts or other musical events? 3. Go to art museums? 4. Go to science museums? 5. Go to history museums? | 0 no  1 yes | 1 | 0.66 |
| **Reportedly antisocial and criminalized behavior (RACB)**  During the first semester of the current school year, has any of the following things happened to you?   1. I was sent to the office because I was misbehaving (wave 1) / I got into trouble for not following school rules (waves 2-3) 2. My parents received a warning about my behavior (waves 1-2) / Parent reported being contacted about their teenager’s behavior in school (wave 3) 3. I got into a physical fight with another student (wave 1) / I got into a physical fight at school (waves 2-3) | 0 never  1 sometimes   (1-2 times)  2 often   (3+ times) | 1-3 | 1: 0.75  2: 0.60  3: 0.47 |
| **Attitudes towards RACB**  How often do you feel it is ‘OK’ for you to:   1. Get into physical fights? 2. Belong to gangs? 3. Make racist remarks? 4. Make sexist remarks? 5. Steal belongings from school, a student, or a teacher? 6. Destroy or damage school property? 7. Smoke on school grounds? 8. Drink alcohol during school day? 9. Use illegal drugs during school day? 10. Bring weapons to school? 11. Abuse teachers physically? 12. Talk back to teachers? | 0 never  1 rarely  2 sometimes  3 often | 2 | 0.81 |

**Table S3.** *Characteristics of adolescents in both cohorts who were excluded from this study* (*those who completed wave 1 but not waves 2 and 3) in comparison to adolescents who were included in the final sample (who completed all 3 waves).*

|  | **Add Health** | | | **NELS:88** | | |
| --- | --- | --- | --- | --- | --- | --- |
|  | **Excluded**  (n=7,849) | **Included**  (n=10,106) | **p value** | **Excluded**  (n=6,884) | **Included**  (n=15,214) | **p value** |
|  | Mean (SD) and t-tests | | | | | |
| Age (years) | 16.24 (1.76) | 15.32 (1.61) | <0.001 | - | - | - |
| Arts total | 1.53 (1.14) | 1.69 (1.19) | <0.001 | 5.00 (2.61) | 5.19 (2.60) | <0.001 |
| RACBs total | 1.59 (3.03) | 1.53 (2.86) | 0.127 | 1.17 (1.37) | 0.93 (1.25) | <0.001 |
| Self-control total | 9.11 (4.68) | 9.16 (4.67) | 0.487 | - | - | - |
|  | Proportion and chi-square tests | | | | | |
| Age (groups) |  |  |  |  |  |  |
| ≤13 years | - | - | - | 1% | 1% | <0.001 |
| 14 years | - | - |  | 61% | 65% |  |
| 15 years | - | - |  | 31% | 29% |  |
| ≥16 years | - | - |  | 7% | 5% |  |
| Gender |  |  |  |  |  |  |
| Male | 52% | 47% | <0.001 | 50% | 49% | 0.184 |
| Female | 48% | 53% |  | 50% | 51% |  |
| Race/ethnicity |  |  |  |  |  |  |
| White | 59% | 62% | <0.001 | 61% | 72% | <0.001 |
| Black | 23% | 21% |  | 18% | 10% |  |
| Asian/Pacific Islander | 7% | 7% |  | 6% | 6% |  |
| Other | 11% | 10% |  | 15% | 12% |  |
| First language |  |  |  |  |  |  |
| English | 87% | 90% | <0.001 | 87% | 89% | <0.001 |
| Other | 13% | 10% |  | 13% | 11% |  |
| Urbanicity |  |  |  |  |  |  |
| Urban | 41% | 36% | <0.001 | 41% | 26% | <0.001 |
| Suburban | 36% | 37% |  | 40% | 42% |  |
| Rural | 23% | 27% |  | 19% | 32% |  |
| Parental education |  |  |  |  |  |  |
| Less than high school | 14% | 12% | <0.001 | 11% | 10% | <0.001 |
| High school | 31% | 30% |  | 19% | 19% |  |
| Some college | 22% | 20% |  | 42% | 40% |  |
| College graduate | 33% | 38% |  | 28% | 31% |  |
| Parental marital status |  |  |  |  |  |  |
| Married | 68% | 73% | <0.001 | 74% | 82% | <0.001 |
| Unmarried | 32% | 27% |  | 26% | 18% |  |
| Household income (quartiles) |  |  |  |  |  |  |
| 1 | 27% | 23% | <0.001 | 33% | 26% | <0.001 |
| 2 | 25% | 26% |  | 26% | 28% |  |
| 3 | 28% | 29% |  | 18% | 21% |  |
| 4 | 20% | 22% |  | 23% | 25% |  |

*Note.* Results are not weighted, and so may differ to Table 1 in the manuscript. In Add Health, some participants were missing data on parental education (excluded 7%, included 5%), parental marital status (excluded 19%, included 7%), and household income (excluded 30%, included 18%). In NELS:88, some participants were missing data on parental education (excluded 1%, included <1%), parental marital status (excluded 4%, included 2%), and household income (excluded 6%, included 4%). Household income quartiles differed slightly across cohorts (Add Health $0-$20,000, $21,000-$38,000, $39,000-$60,000, $61,000+; NELS:88 $0-$19,999, $20,000-$34,999, $35,000-$49,999, $50,000+).

**Table S4.** *Standardized results from the structural equation model in the Add Health cohort.*

|  | **Coef.** | **95% CI** | **p value** |
| --- | --- | --- | --- |
| ARTS & CULTURAL ENGAGEMENT |  |  |  |
| SEP | **-0.19** | **-0.23 to -0.15** | **<0.001** |
| Age | **-0.25** | **-0.28 to -0.22** | **<0.001** |
| Gender | 0.04 | 0.01 to 0.07 | 0.057 |
| First language | **-0.09** | **-0.12 to -0.05** | **<0.001** |
| Race/ethnicity: Black | **-0.07** | **-0.11 to -0.03** | **0.002** |
| Race/ethnicity: Asian/Pacific Islander | -0.02 | -0.04 to 0.01 | 0.270 |
| Race/ethnicity: Other | -0.03 | -0.07 to 0.01 | 0.164 |
| Urbanicity: suburban | 0.05 | 0.01 to 0.09 | 0.050 |
| Urbanicity: rural | -0.01 | -0.06 to 0.03 | 0.622 |
| WAVE 1 BEHAVIOR |  |  |  |
| ARTS & CULTURAL ENGAGEMENT | **-0.11** | **-0.15 to -0.07** | **<0.001** |
| SEP | **0.10** | **0.06 to 0.14** | **<0.001** |
| Age | 0.00 | -0.04 to 0.03 | 0.847 |
| Gender | **-0.26** | **-0.29 to -0.23** | **<0.001** |
| First language | **-0.08** | **-0.10 to -0.05** | **<0.001** |
| Race/ethnicity: Black | **0.07** | **0.04 to 0.10** | **<0.001** |
| Race/ethnicity: Asian/Pacific Islander | 0.02 | -0.01 to 0.04 | 0.272 |
| Race/ethnicity: Other | **0.10** | **0.07 to 0.14** | **<0.001** |
| Urbanicity: suburban | -0.02 | -0.06 to 0.01 | 0.290 |
| Urbanicity: rural | **-0.05** | **-0.09 to -0.01** | **0.051** |
| WAVE 2 BEHAVIOR |  |  |  |
| ARTS & CULTURAL ENGAGEMENT | **-0.06** | **-0.1 to -0.03** | **0.007** |
| WAVE 1 SELF-CONTROL | **0.75** | **0.71 to 0.79** | **<0.001** |
| WAVE 1 BEHAVIOR | -0.02 | -0.06 to 0.01 | 0.234 |
| SEP | 0.04 | 0.00 to 0.07 | 0.063 |
| Age | **-0.09** | **-0.12 to -0.07** | **<0.001** |
| Gender | -0.03 | -0.06 to 0.00 | 0.118 |
| First language | -0.02 | -0.05 to 0.01 | 0.354 |
| Race/ethnicity: Black | -0.01 | -0.04 to 0.02 | 0.473 |
| Race/ethnicity: Asian/Pacific Islander | -0.02 | -0.04 to 0.01 | 0.197 |
| Race/ethnicity: Other | 0.00 | -0.03 to 0.04 | 0.820 |
| Urbanicity: suburban | -0.01 | -0.04 to 0.03 | 0.781 |
| Urbanicity: rural | -0.02 | -0.05 to 0.02 | 0.499 |
| WAVE 3 BEHAVIOR |  |  |  |
| ARTS & CULTURAL ENGAGEMENT | 0.03 | -0.01 to 0.08 | 0.244 |
| WAVE 2 SELF-CONTROL | **0.04** | **0.01 to 0.07** | **0.028** |
| WAVE 2 BEHAVIOR | **0.42** | **0.37 to 0.46** | **<0.001** |
| SEP | **-0.07** | **-0.12 to -0.03** | **0.007** |
| Age | **-0.15** | **-0.18 to -0.12** | **<0.001** |
| Gender | **-0.30** | **-0.33 to -0.27** | **<0.001** |
| First language | -0.02 | -0.05 to 0.02 | 0.431 |
| Race/ethnicity: Black | **0.08** | **0.05 to 0.11** | **<0.001** |
| Race/ethnicity: Asian/Pacific Islander | -0.01 | -0.04 to 0.02 | 0.542 |
| Race/ethnicity: Other | 0.00 | -0.03 to 0.03 | 0.932 |
| Urbanicity: suburban | 0.02 | -0.02 to 0.06 | 0.405 |
| Urbanicity: rural | 0.00 | -0.03 to 0.04 | 0.849 |
| WAVE 1 SELF-CONTROL |  |  |  |
| ARTS & CULTURAL ENGAGEMENT | **-0.20** | **-0.24 to -0.17** | **<0.001** |
| SEP | **0.07** | **0.03 to 0.10** | **0.001** |
| Age | **0.05** | **0.03 to 0.08** | **0.001** |
| Gender | **0.06** | **0.04 to 0.09** | **<0.001** |
| First language | **-0.09** | **-0.12 to -0.06** | **<0.001** |
| Race/ethnicity: Black | **-0.09** | **-0.11 to -0.06** | **<0.001** |
| Race/ethnicity: Asian/Pacific Islander | -0.02 | -0.04 to 0.01 | 0.287 |
| Race/ethnicity: Other | 0.02 | 0.00 to 0.05 | 0.142 |
| Urbanicity: suburban | 0.01 | -0.02 to 0.03 | 0.741 |
| Urbanicity: rural | 0.00 | -0.04 to 0.03 | 0.849 |
| WAVE 2 SELF-CONTROL |  |  |  |
| ARTS & CULTURAL ENGAGEMENT | **0.04** | **0.01 to 0.07** | **0.024** |
| WAVE 1 SELF-CONTROL | **0.74** | **0.72 to 0.76** | **<0.001** |
| WAVE 1 BEHAVIOR | **0.05** | **0.02 to 0.09** | **0.017** |
| SEP | 0.00 | -0.03 to 0.02 | 0.832 |
| Age | **-0.04** | **-0.06 to -0.01** | **0.012** |
| Gender | 0.01 | -0.01 to 0.04 | 0.417 |
| First language | 0.01 | -0.01 to 0.03 | 0.520 |
| Race/ethnicity: Black | -0.02 | -0.03 to 0.00 | 0.151 |
| Race/ethnicity: Asian/Pacific Islander | **0.02** | **0.00 to 0.04** | **0.041** |
| Race/ethnicity: Other | 0.03 | 0.00 to 0.05 | 0.095 |
| Urbanicity: suburban | 0.02 | 0.00 to 0.05 | 0.121 |
| Urbanicity: rural | -0.01 | -0.03 to 0.02 | 0.730 |
| **Covariance** |  |  |  |
| WAVE 1 SELF-CONTROL & BEHAVIOR | **0.45** | **0.42 to 0.47** | **<0.001** |
| WAVE 2 SELF-CONTROL & BEHAVIOR | **0.25** | **0.20 to 0.30** | **<0.001** |

*Note.* Capitalization indicates latent variables. SEP: socioeconomic position. On each latent factor, higher scores indicated more arts and cultural engagement, more behavior, worse self-control, or lower socioeconomic position. Reference categories were male for gender, English for first language, White for race/ethnicity, and urban for urbanicity. See Figure S1 for the standardized factor loadings for each latent variable. Standardized coefficients can be interpreted as the change in the outcome (in outcome standard deviation units) for a standard deviation change in the exposure. Bold text indicates p<0.05.

**Table S5.** *Standardized results from the structural equation model in the NELS:88 cohort.*

|  | **Coef.** | **95% CI** | **p value** |
| --- | --- | --- | --- |
| ARTS & CULTURAL ENGAGEMENT |  |  |  |
| SEP | **0.45** | **0.42 to 0.47** | **<0.001** |
| Gender | **0.10** | **0.08 to 0.12** | **<0.001** |
| First language | **-0.04** | **-0.06 to -0.01** | **0.035** |
| Age: 14 years | -0.05 | -0.24 to 0.14 | 0.652 |
| Age: 15 years | -0.16 | -0.34 to 0.02 | 0.140 |
| Age: ≥16 years | **-0.17** | **-0.26 to -0.08** | **0.002** |
| Race/ethnicity: Black | **-0.11** | **-0.14 to -0.07** | **<0.001** |
| Race/ethnicity: Asian/Pacific Islander | **0.03** | **0.02 to 0.05** | **0.002** |
| Race/ethnicity: Other | **-0.11** | **-0.14 to -0.09** | **<0.001** |
| Urbanicity: suburban | -0.04 | -0.08 to -0.01 | 0.059 |
| Urbanicity: rural | **-0.21** | **-0.24 to -0.17** | **<0.001** |
| WAVE 1 BEHAVIOR |  |  |  |
| ARTS & CULTURAL ENGAGEMENT | **-0.11** | **-0.16 to -0.07** | **<0.001** |
| SEP | **-0.12** | **-0.18 to -0.07** | **<0.001** |
| Gender | **-0.35** | **-0.37 to -0.32** | **<0.001** |
| First language | -0.01 | -0.04 to 0.02 | 0.707 |
| Age: 14 years | 0.04 | -0.22 to 0.29 | 0.816 |
| Age: 15 years | 0.16 | -0.09 to 0.41 | 0.292 |
| Age: ≥16 years | 0.12 | -0.01 to 0.24 | 0.121 |
| Race/ethnicity: Black | **0.11** | **0.08 to 0.15** | **<0.001** |
| Race/ethnicity: Asian/Pacific Islander | **-0.04** | **-0.06 to -0.02** | **0.002** |
| Race/ethnicity: Other | **0.04** | **0.01 to 0.07** | **0.013** |
| Urbanicity: suburban | 0.00 | -0.05 to 0.04 | 0.878 |
| Urbanicity: rural | -0.03 | -0.08 to 0.02 | 0.334 |
| WAVE 2 BEHAVIOR |  |  |  |
| ARTS & CULTURAL ENGAGEMENT | 0.01 | -0.04 to 0.05 | 0.814 |
| WAVE 1 BEHAVIOR | **0.70** | **0.66 to 0.74** | **<0.001** |
| SEP | -0.01 | -0.07 to 0.04 | 0.653 |
| Gender | **-0.10** | **-0.13 to -0.06** | **<0.001** |
| First language | 0.01 | -0.02 to 0.04 | 0.578 |
| Age: 14 years | **0.25** | **0.04 to 0.45** | **0.049** |
| Age: 15 years | 0.21 | 0.01 to 0.41 | 0.079 |
| Age: ≥16 years | 0.10 | 0.00 to 0.20 | 0.104 |
| Race/ethnicity: Black | -0.04 | -0.07 to 0.00 | 0.094 |
| Race/ethnicity: Asian/Pacific Islander | **-0.04** | **-0.06 to -0.02** | **0.007** |
| Race/ethnicity: Other | -0.02 | -0.05 to 0.01 | 0.289 |
| Urbanicity: suburban | 0.01 | -0.04 to 0.06 | 0.698 |
| Urbanicity: rural | -0.01 | -0.06 to 0.05 | 0.872 |
| WAVE 3 BEHAVIOR |  |  |  |
| ARTS & CULTURAL ENGAGEMENT | 0.00 | -0.05 to 0.05 | 0.953 |
| WAVE 2 ATTITUDES | **-0.18** | **-0.25 to -0.12** | **<0.001** |
| WAVE 2 BEHAVIOR | **0.98** | **0.91 to 1.05** | **<0.001** |
| SEP | 0.02 | -0.04 to 0.07 | 0.604 |
| Gender | **-0.14** | **-0.17 to -0.11** | **<0.001** |
| First language | -0.03 | -0.06 to 0.00 | 0.060 |
| Age: 14 years | -0.09 | -0.21 to 0.03 | 0.211 |
| Age: 15 years | -0.07 | -0.19 to 0.05 | 0.316 |
| Age: ≥16 years | -0.01 | -0.07 to 0.05 | 0.774 |
| Race/ethnicity: Black | 0.02 | -0.02 to 0.06 | 0.469 |
| Race/ethnicity: Asian/Pacific Islander | 0.02 | -0.01 to 0.04 | 0.298 |
| Race/ethnicity: Other | 0.01 | -0.02 to 0.04 | 0.538 |
| Urbanicity: suburban | 0.03 | -0.02 to 0.07 | 0.345 |
| Urbanicity: rural | 0.04 | -0.01 to 0.08 | 0.182 |
| WAVE 2 ATTITUDES |  |  |  |
| ARTS & CULTURAL ENGAGEMENT | **-0.06** | **-0.10 to -0.02** | **0.015** |
| WAVE 1 BEHAVIOR | **0.41** | **0.37 to 0.45** | **<0.001** |
| SEP | 0.05 | 0.01 to 0.09 | 0.070 |
| Gender | **-0.16** | **-0.19 to -0.13** | **<0.001** |
| First language | -0.01 | -0.03 to 0.02 | 0.646 |
| Age: 14 years | 0.16 | -0.02 to 0.35 | 0.149 |
| Age: 15 years | 0.09 | -0.09 to 0.27 | 0.401 |
| Age: ≥16 years | 0.02 | -0.07 to 0.11 | 0.684 |
| Race/ethnicity: Black | **-0.14** | **-0.17 to -0.11** | **<0.001** |
| Race/ethnicity: Asian/Pacific Islander | -0.01 | -0.03 to 0.01 | 0.322 |
| Race/ethnicity: Other | **-0.05** | **-0.08 to -0.02** | **0.002** |
| Urbanicity: suburban | 0.03 | -0.01 to 0.07 | 0.174 |
| Urbanicity: rural | 0.03 | -0.02 to 0.07 | 0.332 |
| **Covariance** |  |  |  |
| WAVE 2 ATTITUDES & BEHAVIOR | **0.66** | **0.62 to 0.69** | **<0.001** |

*Note.* Capitalization indicates latent variables. SEP: socioeconomic position. On each latent factor, higher scores indicated more arts and cultural engagement, more RACBs, more positive perceptions of RACBs, or higher socioeconomic position. Reference categories were male for gender, English for first language, ≤13 years for age, White for race/ethnicity, and urban for urbanicity. See Figure S2 for the standardized factor loadings for each latent variable. Standardized coefficients can be interpreted as the change in the outcome (in outcome standard deviation units) for a standard deviation change in the exposure. Bold text indicates p<0.05.

**Table S6.** *Standardized effects of interest from the full structural equation models in both cohorts, modelled separately by gender.*

|  | **Males** | | | **Females** | | |
| --- | --- | --- | --- | --- | --- | --- |
|  | Coef. | 95% CI | p value | Coef. | 95% CI | p value |
| **Add Health** | | | | | | |
| ARTS & CULTURAL ENGAGEMENT 🡪 WAVE 1 SELF-CONTROL | | | |  |  |  |
| Direct: C1 – S1 | **-0.17** | **-0.22 to -0.12** | **<0.001** | **-0.24** | **-0.28 to -0.19** | **<0.001** |
| ARTS & CULTURAL ENGAGEMENT 🡪 WAVE 1 BEHAVIOR | | | |  |  |  |
| Direct: C1 – B1 | **-0.11** | **-0.16 to -0.06** | **<0.001** | **-0.16** | **-0.22 to -0.11** | **<0.001** |
| ARTS & CULTURAL ENGAGEMENT 🡪 WAVE 2 SELF-CONTROL | | | |  |  |  |
| Total | **-0.06** | **-0.12 to -0.01** | **0.043** | **-0.17** | **-0.22 to -0.12** | **<0.001** |
| Total indirect | **-0.13** | **-0.17 to -0.10** | **<0.001** | **-0.18** | **-0.22 to -0.15** | **<0.001** |
| Indirect: C1 – S1 – S2 | **-0.13** | **-0.17 to -0.09** | **<0.001** | **-0.18** | **-0.21 to -0.14** | **<0.001** |
| Indirect: C1 – B1 – S2 | -0.01 | -0.01 to 0.00 | 0.141 | -0.01 | -0.01 to 0.00 | 0.148 |
| Direct: C1 – S2 | **0.07** | **0.03 to 0.11** | **0.003** | 0.01 | -0.03 to 0.06 | 0.555 |
| ARTS & CULTURAL ENGAGEMENT 🡪 WAVE 2 BEHAVIOR | | | |  |  |  |
| Total | -0.06 | -0.11 to 0.00 | 0.091 | **-0.16** | **-0.21 to -0.1** | **<0.001** |
| Total indirect | **-0.08** | **-0.12 to -0.04** | **0.001** | **-0.12** | **-0.16 to -0.08** | **<0.001** |
| Indirect: C1 – S1 – B2 | 0.00 | -0.01 to 0.01 | 0.701 | 0.01 | 0.00 to 0.02 | 0.283 |
| Indirect: C1 – B1 – B2 | **-0.08** | **-0.12 to -0.04** | **<0.001** | **-0.13** | **-0.17 to -0.09** | **<0.001** |
| Direct: C1 – B2 | 0.02 | -0.03 to 0.07 | 0.440 | -0.03 | -0.09 to 0.02 | 0.292 |
| ARTS & CULTURAL ENGAGEMENT 🡪 WAVE 3 BEHAVIOR | | | |  |  |  |
| Total | **-0.08** | **-0.14 to -0.02** | **0.040** | 0.01 | -0.06 to 0.08 | 0.758 |
| Total indirect | -0.03 | -0.05 to 0.00 | 0.078 | **-0.08** | **-0.11 to -0.06** | **<0.001** |
| Indirect: C1 – S2 – B3 | 0.00 | 0.00 to 0.01 | 0.456 | 0.00 | 0.00 to 0.01 | 0.562 |
| Indirect: C1 – B2 – B3 | 0.01 | -0.01 to 0.03 | 0.443 | -0.02 | -0.04 to 0.01 | 0.292 |
| Indirect: C1 – S1 – S2 – B3 | 0.00 | -0.01 to 0.00 | 0.434 | **-0.02** | **-0.03 to -0.01** | **0.009** |
| Indirect: C1 – B1 – S2 – B3 | 0.00 | 0.00 to 0.00 | 0.492 | 0.00 | 0.00 to 0.00 | 0.165 |
| Indirect: C1 – S1 – B2 – B3 | 0.00 | 0.00 to 0.00 | 0.702 | 0.00 | 0.00 to 0.01 | 0.292 |
| Indirect: C1 – B1 – B2 – B3 | **-0.04** | **-0.06 to -0.02** | **0.001** | **-0.06** | **-0.08 to -0.04** | **<0.001** |
| Direct: C1 – B3 | -0.05 | -0.11 to 0.01 | 0.181 | **0.10** | **0.03 to 0.17** | **0.025** |
| **NELS:88** | | | | | | |
| ARTS & CULTURAL ENGAGEMENT 🡪 WAVE 1 BEHAVIOR | | | |  |  |  |
| Direct: C1 – B1 | **-0.09** | **-0.14 to -0.03** | **0.009** | **-0.13** | **-0.18 to -0.07** | **<0.001** |
| ARTS & CULTURAL ENGAGEMENT 🡪 WAVE 2 ATTITUDES | | | |  |  |  |
| Total | **-0.08** | **-0.12 to -0.03** | **0.010** | **-0.11** | **-0.15 to -0.07** | **<0.001** |
| Total indirect | **-0.06** | **-0.09 to -0.04** | **<0.001** | -0.02 | -0.03 to 0.00 | 0.091 |
| Indirect: C1 – B1 – A2 | **-0.06** | **-0.09 to -0.04** | **<0.001** | -0.02 | -0.03 to 0.00 | 0.091 |
| Direct: C1 – A2 | -0.01 | -0.06 to 0.04 | 0.704 | **-0.09** | **-0.13 to -0.06** | **<0.001** |
| ARTS & CULTURAL ENGAGEMENT 🡪 WAVE 2 BEHAVIOR | | | |  |  |  |
| Total | -0.06 | -0.11 to 0.00 | 0.102 | **-0.09** | **-0.15 to -0.03** | **0.010** |
| Total indirect | **-0.11** | **-0.15 to -0.07** | **<0.001** | -0.05 | -0.09 to 0.00 | 0.092 |
| Indirect: C1 – B1 – B2 | **-0.11** | **-0.15 to -0.07** | **<0.001** | -0.05 | -0.09 to 0.00 | 0.092 |
| Direct: C1 – B2 | 0.06 | -0.01 to 0.12 | 0.140 | -0.05 | -0.12 to 0.02 | 0.226 |
| ARTS & CULTURAL ENGAGEMENT 🡪 WAVE 3 BEHAVIOR | | | |  |  |  |
| Total | **-0.11** | **-0.18 to -0.05** | **0.006** | 0.02 | -0.04 to 0.09 | 0.576 |
| Total indirect | -0.04 | -0.09 to 0.01 | 0.172 | -0.06 | -0.12 to 0.00 | 0.098 |
| Indirect: C1 – A2 – B3 | 0.00 | -0.01 to 0.01 | 0.704 | **0.03** | **0.01 to 0.05** | **0.014** |
| Indirect: C1 – B2 – B3 | 0.06 | -0.01 to 0.12 | 0.147 | -0.05 | -0.12 to 0.02 | 0.232 |
| Indirect: C1 – B1 – A2 – B3 | **0.01** | **0.00 to 0.02** | **0.015** | 0.01 | 0.00 to 0.01 | 0.118 |
| Indirect: C1 – B1 – B2 – B3 | **-0.11** | **-0.15 to -0.07** | **<0.001** | -0.05 | -0.09 to 0.00 | 0.089 |
| Direct: C1 – B3 | -0.07 | -0.14 to 0.00 | 0.083 | 0.08 | 0.01 to 0.16 | 0.064 |

*Note.* The number suffix indicates the wave at which each latent variable was measured. C: arts and cultural engagement. B: reportedly antisocial or criminalized behavior. S: self-control scores. A: attitudes towards reportedly antisocial or criminalized behavior. Bold text indicates p<0.05. Standardized coefficients can be interpreted as the change in the outcome (in outcome standard deviation units) for a standard deviation change in the exposure.

**Table S7.** *Standardized effects of interest from the structural equation model in the Add Health cohort including only violent reportedly antisocial or criminalized behavior.*

|  | **Coef.** | **95% CI** | **p value** |
| --- | --- | --- | --- |
| ARTS & CULTURAL ENGAGEMENT 🡪 WAVE 1 SELF-CONTROL | | | |
| Direct: C1 – S1 | **-0.20** | **-0.24 to -0.17** | **<0.001** |
| ARTS & CULTURAL ENGAGEMENT 🡪 WAVE 1 BEHAVIOR | | | |
| Direct: C1 – B1 | **-0.09** | **-0.14 to -0.04** | **0.004** |
| ARTS & CULTURAL ENGAGEMENT 🡪 WAVE 2 SELF-CONTROL | | | |
| Total | **-0.11** | **-0.15 to -0.08** | **<0.001** |
| Total indirect | **-0.15** | **-0.18 to -0.13** | **<0.001** |
| Indirect: C1 – S1 – S2 | **-0.15** | **-0.18 to -0.12** | **<0.001** |
| Indirect: C1 – B1 – S2 | 0.00 | -0.01 to 0.00 | 0.273 |
| Direct: C1 – S2 | **0.04** | **0.01 to 0.07** | **0.018** |
| ARTS & CULTURAL ENGAGEMENT 🡪 WAVE 2 BEHAVIOR | | | |
| Total | **-0.18** | **-0.23 to -0.13** | **<0.001** |
| Total indirect | **-0.06** | **-0.10 to -0.02** | **0.007** |
| Indirect: C1 – S1 – B2 | 0.00 | 0.00 to 0.01 | 0.376 |
| Indirect: C1 – B1 – B2 | **-0.06** | **-0.10 to -0.03** | **0.004** |
| Direct: C1 – B2 | **-0.12** | **-0.16 to -0.07** | **<0.001** |
| ARTS & CULTURAL ENGAGEMENT 🡪 WAVE 3 BEHAVIOR | | | |
| Total | -0.01 | -0.07 to 0.05 | 0.811 |
| Total indirect | **-0.09** | **-0.11 to -0.06** | **<0.001** |
| Indirect: C1 – S2 – B3 | 0.00 | 0.00 to 0.00 | 0.853 |
| Indirect: C1 – B2 – B3 | **-0.06** | **-0.08 to -0.03** | **<0.001** |
| Indirect: C1 – S1 – S2 – B3 | 0.00 | -0.01 to 0.00 | 0.855 |
| Indirect: C1 – B1 – S2 – B3 | 0.00 | 0.00 to 0.00 | 0.857 |
| Indirect: C1 – S1 – B2 – B3 | 0.00 | 0.00 to 0.01 | 0.379 |
| Indirect: C1 – B1 – B2 – B3 | **-0.03** | **-0.05 to -0.01** | **0.005** |
| Direct: C1 – B3 | **0.08** | **0.02 to 0.13** | **0.022** |

*Note.* The number suffix indicates the wave at which each latent variable was measured. C: arts and cultural engagement. B: violent reportedly antisocial or criminalized behavior. S: self-control scores. Bold text indicates p<0.05. Standardized coefficients can be interpreted as the change in the outcome (in outcome standard deviation units) for a standard deviation change in the exposure.


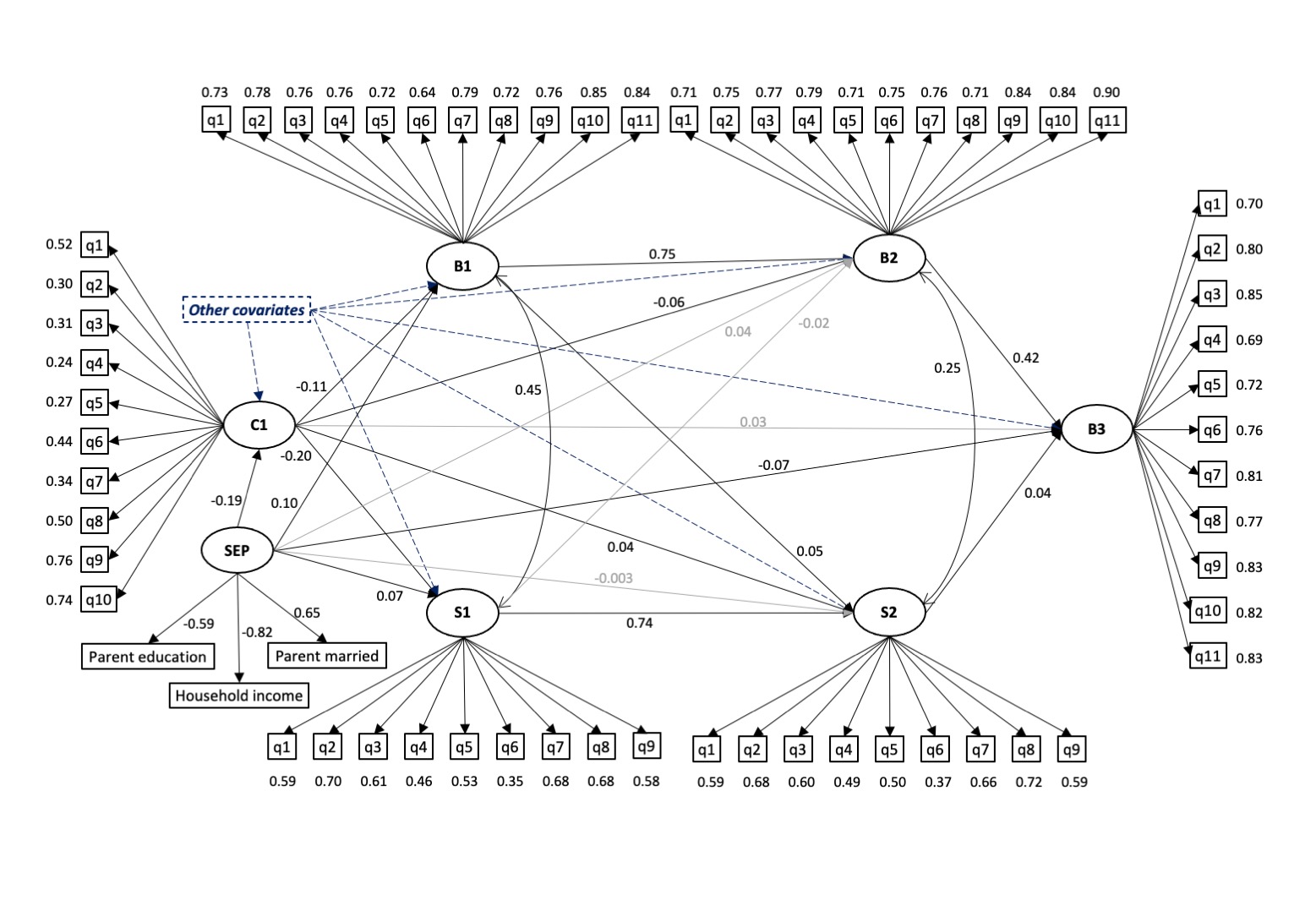


**Figure S1.** Full structural equation model in the Add Health cohort. The number suffix indicates the wave at which each latent variable was measured. Standardized coefficients are presented and those in grey did not reach significance (p>0.05). Standardized coefficients can be interpreted as the change in the outcome (in outcome standard deviation units) for a standard deviation change in the exposure. Factor loadings were allowed to vary across years as constraining them significantly worsened model fit. C: arts and cultural engagement. B: reportedly antisocial or criminalized behavior. SC: self-control score. SEP: socioeconomic position. Covariates not shown were age, gender, first language, race/ethnicity, and urbanicity (see Table S3 for these results). For all latent factors except SEP, factor loadings are shown to the left/right of (or above/below) the indicator variables for clarity.

**
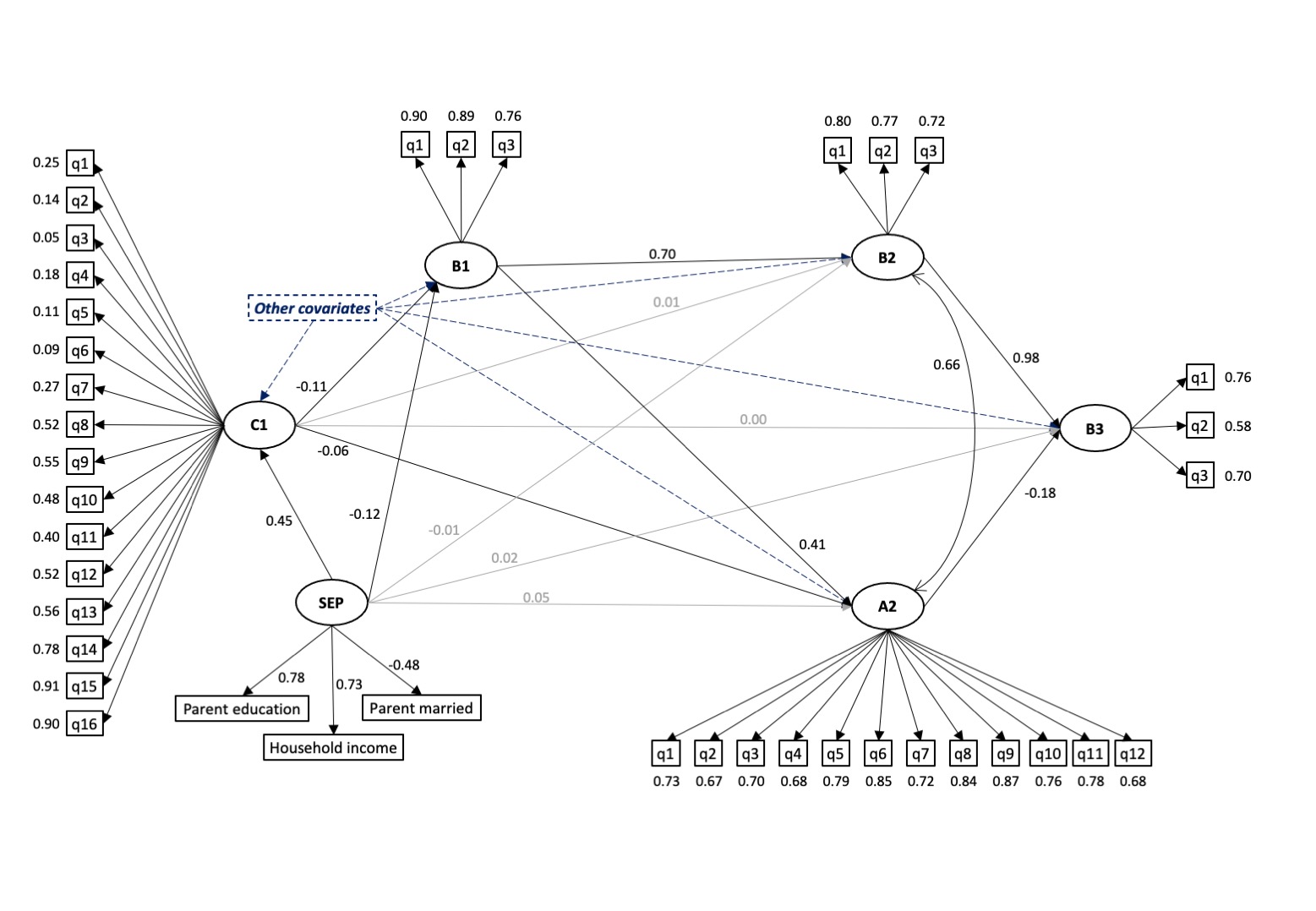
**

**Figure S2.** Full structural equation model in the NELS:88 cohort. The number suffix indicates the wave at which each latent variable was measured. Standardized coefficients are presented and those in grey did not reach significance (p>0.05). Standardized coefficients can be interpreted as the change in the outcome (in outcome standard deviation units) for a standard deviation change in the exposure. Factor loadings were allowed to vary across years as constraining them significantly worsened model fit. C: arts and cultural engagement. B: reportedly antisocial or criminalized behavior. A: attitudes towards reportedly antisocial and criminalized behavior. SEP: socioeconomic position. Covariates not shown were age, gender, first language, race/ethnicity, and urbanicity (see Table S4 for these results). For all latent factors except SEP, factor loadings are shown to the left/right of (or above/below) the indicator variables for clarity.
